# Community-based surveillance: A scoping review

**DOI:** 10.1371/journal.pone.0215278

**Published:** 2019-04-12

**Authors:** José Guerra, Pratikshya Acharya, Céline Barnadas

**Affiliations:** World Health Organization (WHO), Lyon, France; University of Queensland, AUSTRALIA

## Abstract

**Background:**

Involving community members in identifying and reporting health events for public health surveillance purposes, an approach commonly described as community-based surveillance (CBS), is increasingly gaining interest. We conducted a scoping review to list terms and definitions used to characterize CBS, to identify and summarize available guidance and recommendations, and to map information on past and existing in-country CBS systems.

**Methods:**

We searched eight bibliographic databases and screened the worldwide web for any document mentioning an approach in which community members both collected and reported information on health events from their community for public health surveillance. Two independent reviewers performed double blind screening and data collection, any discrepancy was solved through discussion and consensus.

**Findings:**

From the 134 included documents, several terms and definitions for CBS were retrieved. Guidance and recommendations for CBS were scattered through seven major guides and sixteen additional documents. Seventy-nine unique CBS systems implemented since 1958 in 42 countries were identified, mostly implemented in low and lower-middle income countries (79%). The systems appeared as fragmented (81% covering a limited geographical area and 70% solely implemented in a rural setting), vertical (67% with a single scope of interest), and of limited duration (median of 6 years for ongoing systems and 2 years for ended systems). Collection of information was mostly performed by recruited community members (80%).

**Interpretation:**

While CBS has already been implemented in many countries, standardization is still required on the term and processes to be used. Further research is needed to ensure CBS integrates effectively into the overall public health surveillance system.

## Introduction

Public health surveillance is an essential function of a health system, defined as **“***the systematic on-going collection*, *collation and analysis of data for public health purposes and the timely dissemination of public health information for assessment and public health response as necessar*y” [1].

Conventionally, public health surveillance relies on healthcare facilities where information is captured from in- and outpatients [2]. However, it has been suggested that only a portion of sick individuals visit healthcare facilities [3–8], due to unavailability or inaccessibility of health facilities [6]; a reliance on self or alternative medication [8]; or an assumption that disease condition is not serious enough to seek treatment [6,7]. Therefore, to complement healthcare facility-based surveillance, another approach is to involve community members in identifying and reporting health events occurring in their community.

This approach was the topic of a 2001 handbook for community surveillance coordinators published to “encourage the involvement of communities themselves both in detecting and reporting diseases and in preventing disease and promoting positive health habits” [9]. Involvement of communities was also part of the 2001 technical guidelines for integrated disease surveillance and response used in the African region which sought to: “emphasize community participation in detection and response to public health problems” [10]. In its 2010 edition, the term “community-based surveillance” was introduced and a definition provided: “trained surveillance informants identify and report events in the community that have public health significance” [11]. In 2014 and 2015, the World Health Organization published a “guide for establishing community-based surveillance” and a dedicated training manual [12,13]. This was followed by a guidance document on “community-based surveillance” published by the International Federation of Red Cross and Red Crescent Societies in 2017 [14]. These additional guides provided much needed support to involve community members in the approach of identifying and reporting health events occurring in their community. However, certain discrepancies were seen between the guides and information on certain aspects of such an approach were missing. Furthermore, the occurrence of the term “community-based surveillance” in the literature increased, but it was often used to characterize very different approaches. For example, while some documents used the term to describe the involvement of community members for public health surveillance [4], others used it to describe studies performed in healthcare facilities by dedicated surveyors for research purposes [15].

Overall, there is a lack of standardization of the approach involving community members in identifying and reporting health events occurring in their community for public health surveillance (hereafter designated by the acronym CBS), namely a consensual term and definition to characterize it, and the actors and processes involved in its implementation and operation.

In order to support the further standardization of CBS, a scoping review was conducted to systematically list terms and definitions used to characterize CBS, to identify and summarize guidance documents and recommendations available for its implementation and operation, and to map the details of any past and existing examples of in-country CBS systems.

## Materials and methods

This scoping review follows the method proposed by Arksey and O’Malley [16] and modified by Levac [17]. The protocol of the study was not registered.

### Eligibility criteria

In this scoping review, we defined our inclusion criteria as any document mentioning an approach or system with the following characteristics:

- collection of information from the community performed by community members, and
- reporting of this information for public health surveillance purposes (i.e. monitoring of the health status of a population or early detection of public health risks and events).

We defined a community as people living in a defined geographical area.

The following exclusion criteria were applied:

- ad hoc prevalence or incidence study for a specific condition;
- description of an approach or system solely involving collection of information from healthcare facilities;

- language other than English, French, Spanish or Portuguese;
- full text unavailable; or
- conference presentation.

No publication time limit was used for the selection of the documents.

### Search of information sources

A search for eligible documents was conducted using the eight following bibliographic databases: Medline, Global Index Medicus, Popline, Cochrane library, Excerpta Medica database (EMBASE), Iris, The European Library, and Africabib.

The search strategy was designed to identify documents including: both concepts of community participation and public health surveillance, or terms denoting CBS. Tailored search requests were used to select documents from each bibliographic database. As an example, the search request used for Medline using Pubmed on the 28 March 2017 was: (("sentinel surveillance" [MeSH Terms] OR "population surveillance" [MeSH Terms] OR "public health surveillance" [MeSH Terms] OR surveillance [Title/Abstract] OR "public health surveillance") AND ("Community-Based Participatory Research" [MeSH Terms] OR "Community-Institutional Relations" [MeSH Terms] OR "Community Health Workers" [MeSH Terms] OR "volunteers" [MeSH Terms])) OR "community-based surveillance" [TIAB] OR "participatory surveillance" [TIAB] OR "household surveillance" [TIAB] OR "community based sentinel surveillance" [TIAB] OR "community based health reporting" [TIAB].

Additional searches were also conducted using the Google search engine on the worldwide web, where the 50 first results of each of four search requests were screened for suitability. Detailed search requests and search results from each database and the world-wide web are presented in the S1 Table.

Subsequently, the reference lists of each of the documents found to meet the inclusion criteria were also screened to identify any additional documents of interest.

### Selection of sources of evidence

Two reviewers (JG and PA) independently screened in a blind standardized manner the titles and abstracts of each of the search results using the Rayyan web application [18]. Disagreements between reviewers on inclusion or exclusion were resolved by discussion and consensus. Further exclusion of the documents was performed during the data collection process (i.e. a document could be later excluded based on its full-text review).

### Data charting process and data items

**Fig 1:**
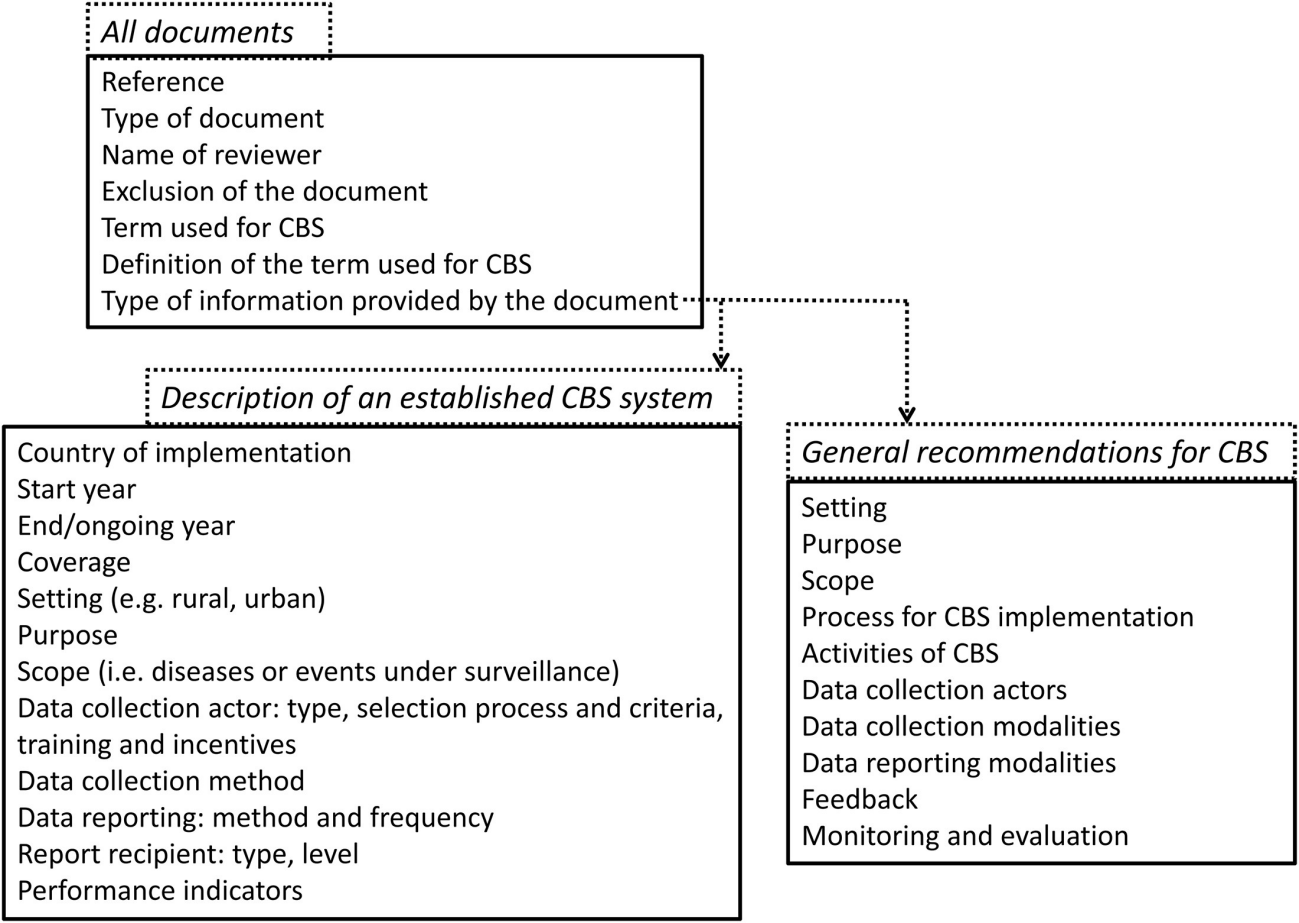
Variables collected from each included document.

We developed a data collection form using the LimeSurvey software [19] to systematically list terms and definitions used to characterize CBS, identify and summarize guidance documents and recommendations available for its implementation and operation, and map the details of any past and existing examples of in-country CBS implementations. The variables collected are listed in Fig 1. The same two reviewers independently filled the data collection form for each included document. Any discrepancy in the collected information was resolved by discussion and consensus.

### Synthesis of results

An analysis of the collected data on terms and definitions used for CBS and past and current examples of in-country CBS systems was performed using the R statistical software [20].

Data collected from different documents were consolidated for each unique CBS system identified.

Evidence tables were developed to present all collected information.

Available guidance and recommendations to implement or operate CBS were summarized.

### Supplemental study on the usage of the term “community-based surveillance”

We conducted a supplemental study on the use of the term “community-based surveillance” in the literature. All unique documents retrieved from the search of information sources were screened anew by one reviewer, the sole inclusion criterion was the explicit mention of the term “community-based” and “surveillance” with or without other elaborative words in between in the title or abstract. Information was collected for the following variables: type of document and description of the approach termed as “community-based surveillance” in the document (full method available in S2 Text).

### Role of the funding source

The funder of the study had no role in study design, data collection, data analysis, data interpretation, or writing of the report. The corresponding author had full access to all the data in the study and had final responsibility for the decision to submit for publication.

## Results

### Selection and characteristics of sources of evidence

**Fig 2:**
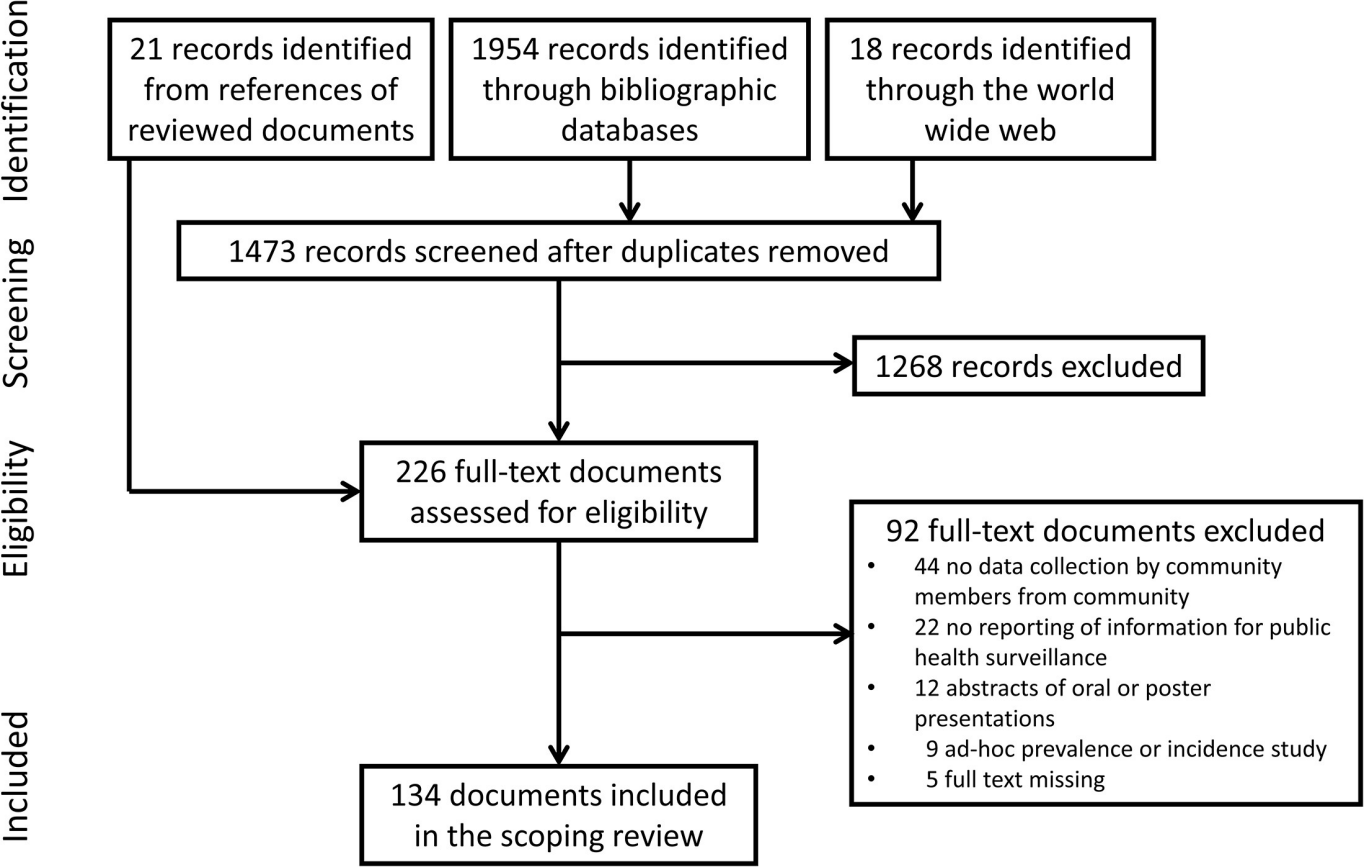
Documents selection.

One thousand nine hundred ninety-three documents were identified by the search strategies. After screening and selection, 134 were included in the review [3–5,9,11–14,21–146] as illustrated below in Fig 2.

The bibliographic reference and type of each included document are available in the S2 Table.

### Terms and definitions used to characterize CBS

Sixty-six percent of documents (n = 88/134) used at least one specific term to denote the approach of involving community members in identifying and reporting health events occurring in their community for the purpose of public health surveillance. The remaining 46 documents mentioned such approach, but without a specific term to denote it. The most commonly used term was “community-based surveillance” (n = 46/88, 52% of documents), followed by “community event-based surveillance” (n = 7/88, 8%). In total, 44 unique terms to denote the concept of CBS were identified. All unique terms comprised two basic components: a component to denote the involvement of community members and a component to denote the concept of public health surveillance. A list of all the terms is available in the S2 Table.

Ten documents contained a specific definition of the term that was used for CBS (7%, n = 10/134) with eight unique definitions retrieved (see Table 1).

**Table 1:** Definitions of the term used to denote the approach of engaging community members in identifying and reporting health events occurring in their community for public health surveillance purposes.

Reference	Definition
**Oum (2005)** [4]	[Community-based surveillance is] “a network of lay people involved in the systematic detection and reporting of health-related events from their community”
**Chau (2007)** [21]	“CBSS [Community-based surveillance system] is a surveillance system which detects and report diseases from within the community by village health volunteers”
**World Health Organization and Centers for Disease Control and Prevention (2010)** [11]	“In this system [Community-based surveillance system], trained surveillance informants identify and report events in the community that have public health significance. Community informants report to the health facility or, in the case of a serious event, directly to the district authorities.”
**Curry (2013)** [22]	“CBS [Community-based surveillance] is a set of activities that increase public awareness of the symptoms of a disease or condition and encourage self-initiated case-reporting by the community to the official MOH [Ministry of Health] and/or WHO surveillance authorities. This system includes a mechanism for active case search in the community by non-clinical volunteers or employees and a system for tracking the cases detected. Two elements of this definition are important to note as central to distinguishing CBS from other forms of active surveillance or outreach: (1) case detection activities occur outside a health facility, and (2) those performing case detection activities are community members.”
**World Health Organization (2014)** [12], **World Health Organization (2015)** [13]	“Community-based Surveillance (CBS) is an active process of community participation in detecting, reporting, responding to and monitoring health events in the community. The scope of CBS is limited to systematic on-going collection of data on events and diseases using simplified case definitions and forms and reporting to health facilities for verification, investigation, collation, analysis and response as necessary. CBS should be a routine function for: (a) the pre-epidemic period (to provide early warning or alerts); (b) the period during epidemic (to actively detect and respond to cases and deaths); (c) the post-epidemic period (to monitor progress with disease control activities). CBS should also include a process to report rumours and misinformation of unusual public health events occurring in the community.”
**Okiror (2015)** [23]	“It [Community-based surveillance] is an ongoing activity conducted at community level by community volunteers and includes active case searches during house-to-house visits, religious and traditional healing sites (holy water, prayers, church, mosque) visits, with kalicha (Muslim traditional healers) and reporting to the nearby health facilities”
**International Rescue Committee (2015)** [24], **Ebola Response Consortum (2015)** [25]	“Community event-based surveillance is the organized and rapid capture of information from the community about events that are a potential risk to public health.”
**International Federation of Red Cross and Red Crescent Societies (2017)** [14]	“Community-based surveillance is a surveillance system that monitors a broad range of information directly from community members. It is a simple, adaptable and low-cost public health initiative managed by communities to protect communities. CBS empowers trained RC [Red Cross/Red Crescent] volunteers to report unusual events in the community where they live through the use of a mobile phone or other form of communication”.

### Available guidance and recommendations for the implementation and operation of CBS

Twenty-three documents (17%) contained guidance material or recommendations related to the planning, implementation or operation of a CBS system [4,9,11–14,22–38]. A summary of available guidance and recommendations is presented in the S1 Text. Seven of these documents were detailed guidance documents with a specific focus on CBS and are presented in Table 2. Many of these documents noted that it was crucial to keep the CBS systems simple, purposeful and easy to set up [14,29,30], with information collected only if it can lead to a response [14,29].

**Table 2:** Major guidance documents on CBS.

Title of the document [ref]	Organization (Year)	Main objective of document	Target audience	Topics addressed
**Handbook for community surveillance coordinators to support community participation in detection and prevention of polio and other diseases** [9]	Academy for Educational Development (2001)	To support polio surveillance for its elimination and surveillance of other diseases, namely: measles, neonatal tetanus, cholera, meningitis, and, yellow fever.	Community surveillance coordinators involved in supervision of surveillance volunteers	**Process of CBS implementation**: roles of different health authority levels and NGOs, modalities to involve community in surveillance program. **Activities of CBS**: roles of different health authority levels and NGOs. **Actors for data collection**: desired qualities, selection modality, ways to motivate them, training modality. **Data collection**: case definitions, samples of reporting forms. **Supervision**, monitoring and evaluation: supervision modality.
**A guide to establishing event-based surveillance** [27]	World Health Organization Regional Office for Western Pacific (2008)	To support the “design of event-based surveillance systems”.	Not specified	**Actors for data collection**: types of actor. **Data collection**: sources of information, list of trigger events, modality. **Data reporting**: modality.
**Integrated disease surveillance and response in the African Region: a guide for establishing community-based surveillance** [12]	World Health Organization Regional Office for Africa (2014)	“To build and strengthen the capacity of communities to conduct effective surveillance and response activities in line with the IDSR [Integrated disease surveillance and response] strategy.“ “To improve the flow of surveillance information between the community and local health facilities.”	Health Facility managers, District Health Management Teams, Health, Education and Agricultural officers, Community-based health workers, National IHR Focal Points, NGOs and other relevant partners such as Red Cross	**Process of CBS implementation**: framework of CBS, steps to implement a CBS system, responsibilities of the local health facility that supervises a CBS system. **Activities of CBS**: activities to be performed under a CBS system, responsibilities of actors for data collection. **Scope**: priority diseases for surveillance. **Actors for data collection**: requirements. **Data collection**: key case definitions, sources of data.
**Ebola and Marburg virus disease epidemics: preparedness, alert, control, and evaluation** [28]	World Health Organization (2014)	“To provide health-care workers in risk areas with a working tool to combat Ebola Virus Disease (EVD) or Marburg Virus Disease (MVD) effectively”	District-level health workers, Intermediate- and central-level health workers responsible for epidemic control, International Health Regulations (IHR) National Focal Points (NFPs)	**Process of CBS implementation**: steps to implement a CBS system.
**Integrated Diseases Surveillance and Response in the African Region. Community-based Surveillance (CBS) Training Manual** [13]	World Health Organization Regional Office for Africa (2015)	To guide training on the aspects of CBS presented in the guide for establishing CBS	Community health workers and anyone who have a role in CBS implementation	**Scope**: priority diseases for surveillance. **Process of CBS implementation**: responsibilities of local health facility that supervises a CBS system. **Actors for data collection**: training topics, training modality. **Data collection**: case definitions of key diseases, three sample forms for reporting. **Data reporting**: modality. **Feedback**: modality to provide feedback to community **Supervision, monitoring and evaluation**: supervision modality, aspects to monitor, aspects to evaluate.
**Standard Operating Procedure for community event-based surveillance for Ebola virus disease in Sierra Leone** [24]	International Rescue Committee (2015)	To describe “the structure and implementation of an effective community event-based surveillance system (CEBS) for Ebola in Sierra Leone” To provide “standardized instruction and protocols for all districts that engage in CEBS”	Community Surveillance Supervisors (CSS), Community Health Monitors (CHMs), Chiefdom Health Officers (CHO), CEBS implementation team, Ministry of Health surveillance officers, District health management team and other key participants at the district and chiefdom level.	**Process of CBS implementation**: steps to implement the CBS system. **Activities of CBS**: procedures/key steps of the system, responsibilities of various actors involved. **Data collection**: list of trigger events for Ebola
**Community-based surveillance: guiding principles** [14]	International Federation of Red Cross and Red Crescent Societies (2017)	“To provide an understanding of CBS and how it can be used in the countries where Red Cross / Red Crescent (RC) volunteers are involved in strengthening existing national surveillance, as well as RC activities”	National RC societies, RC’s health program staffs, Other partner organisations, National authorities, RC volunteers	**Setting**: relevance of a CBS system in a community. **Scope**: considerations to determine scope for surveillance. **Process of CBS implementation**: steps to implement a CBS system, community engagement. **Activities of CBS**: procedure / key steps. **Actors for data collection**: requirement, selection modality, training modality. **Data collection**: desired qualities of triggers and case definitions, modality, tool. **Data reporting**: modality. **Feedback**: modality to communicate with and receive feedback from community people. **Supervision, monitoring and evaluation**: performance indicators.

Several guidance documents provided simplified health events case definitions to be used for CBS, most of which could be found in an available World Health Organization guide for establishing a CBS system [12]. Whilst several documents highlighted the crucial role of feedback in ensuring an effective CBS system, there was almost a total lack of concrete guidance on how to provide such feedback. Similarly, no practical guidance’s or tools were found available to support the proper evaluation of the effectiveness and utility of a CBS system.

### Descriptions of past and existing CBS systems

One hundred fourteen documents (85%) mentioned a past or existing in country-CBS system. From these, 79 unique CBS systems were identified.

The data collected for each unique CBS system is displayed in the S2 Table.

#### Missing information on CBS systems

For each type of variable, the percentage of missing data was (n = 79 unique CBS systems): country 0%; start year 20%; end/ongoing year 22%; coverage 9%; setting 25%; purpose 28%; scope 0%; data collection actor 0%; data collection method 37%; data reporting method 53%, and frequency 24%; report recipient type 28%, and level 32%; performance indicators 75%.

#### Country of implementation

**Fig 3:**
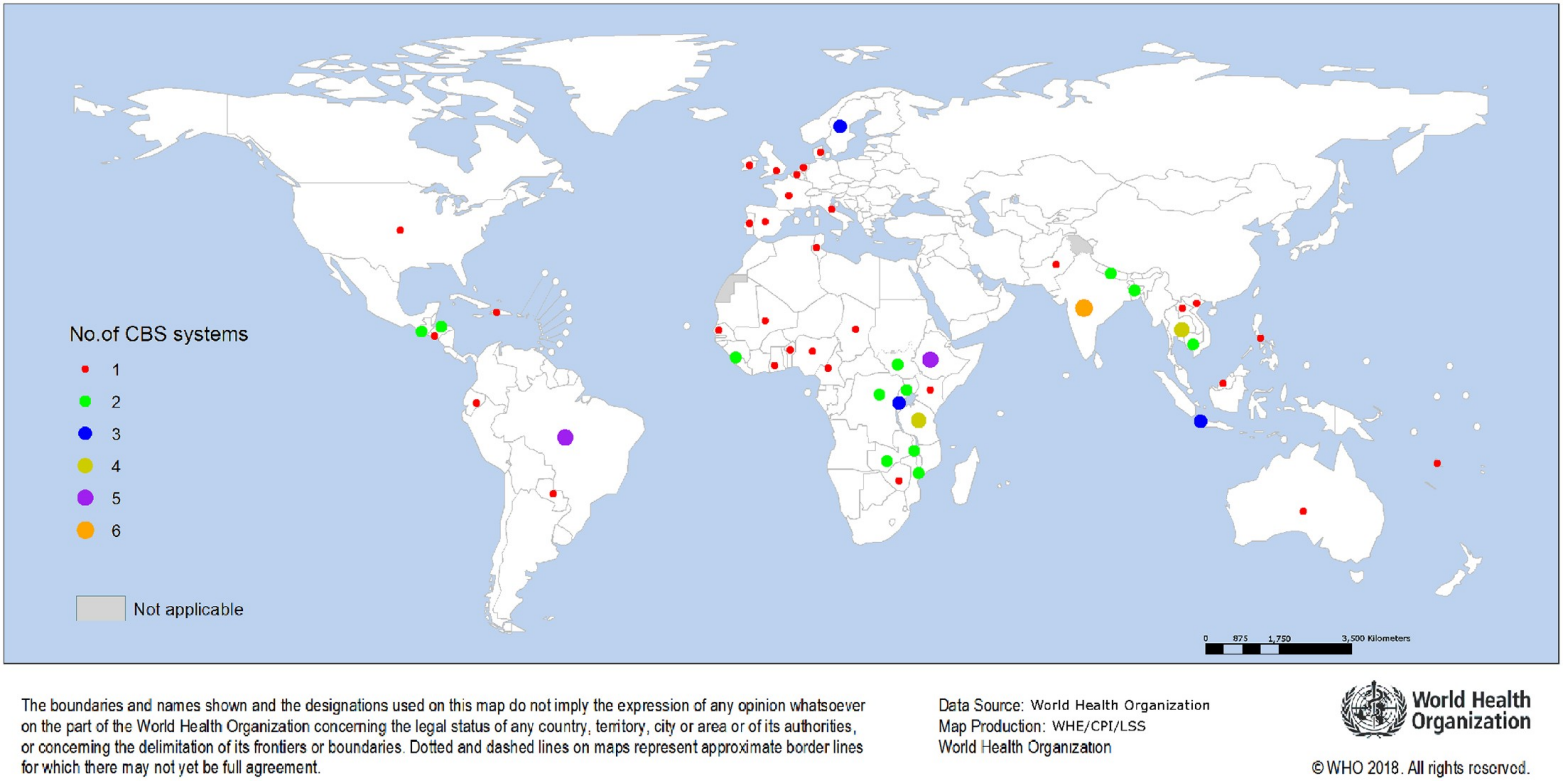
Distribution of CBS systems identified across different countries.

CBS systems were identified in 42 countries (see Fig 3).

#### Operation period

Ninety-two percent of identified CBS systems were established after 1980 (n = 58/63), with an upsurge notable in the period from 2001 to 2010 (45%, n = 28/63). The oldest system was established in 1958 [26]. Sixty-nine percent of systems were described as ongoing (n = 43/62) whilst the remaining 31% (n = 19/62) had ended. The median duration of operation of the ongoing CBS systems was 6 years (IQR [2 years; 13 years], n = 37). The longest-running CBS system was also the oldest, established in Guatemala for malaria surveillance 34 years ago in 1992 [26]. The median duration of operation for the ended CBS systems was 2 years (IQR [1 year; 3 years], n = 19), with a range of 1 month (Democratic Republic of the Congo for a measles outbreak [39]) to 6 years (Tanzania for children’s nutritional status monitoring [40]).

#### Coverage and setting

CBS systems were mostly implemented in limited geographical areas (n = 58/72, 81%) and in the following settings:

- rural (n = 41/59, 70%),
- both rural and urban (n = 10/59, 17%),
- urban (n = 5/59, 8%),
- refugee settlements (n = 3/59, 5%).

#### Purpose

Purposes of the systems were noted as:

- monitor the health status of a population (n = 26/57, 45%),
- early detect public health risks and events (n = 17/57, 30%),
- both above purposes (n = 14/57, 25%).

#### Scope

**Fig 4:**
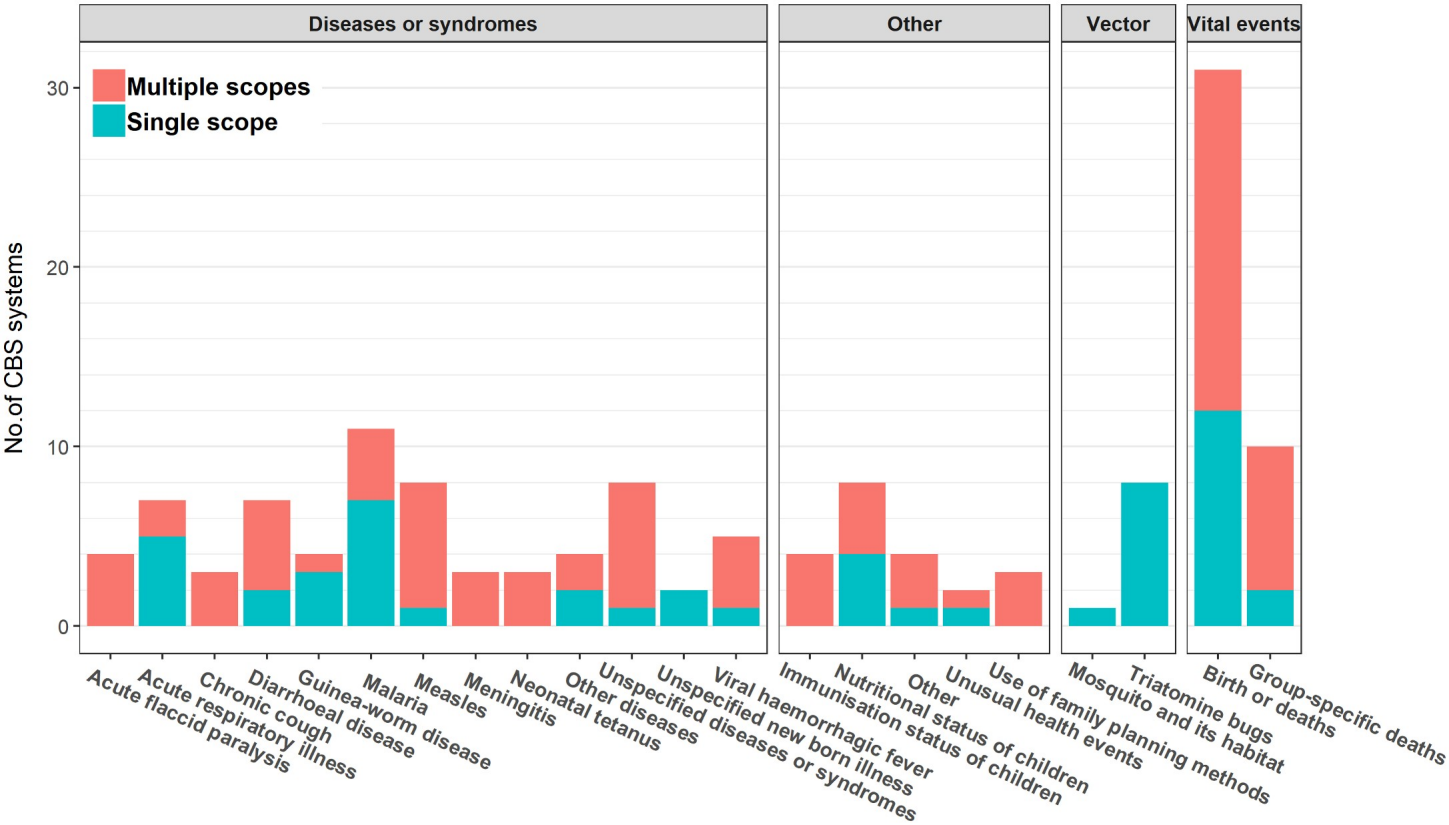
Distribution of CBS systems by scopes of interest (n = 79 systems). ^a^ includes influenza like illness and avian influenza; ^b^ includes cholera, acute gastrointestinal illnesses; ^c^ includes Buruli ulcer (n = 1), cutaneous leishmaniasis (n = 1), yaws (n = 1), smallpox (n = 1); ^d^ includes Ebola virus disease and dengue; ^e^ includes pregnancy complications (n = 2), low birth weight (n = 1), suicidal and self-injurious behaviour (n = 1); ^f^ includes maternal, neonatal, infant, under-five deaths.

The scope of the health events under surveillance are presented in Fig 4. Most systems focused on a single health condition or event (n = 53/79, 67%).

#### Actors in charge of data collection

Three different types of community members were identified in the documents as performing data collection (hereafter named as “data collectors”):

- locally recruited surveillance cadres (i.e. community members who were selected and recruited as volunteers or paid workers): n = 56/79 (71%);
- general community members (i.e. any community member could report an event): n = 12/79 (15%);
- a specified group of the community (i.e. certain group of community members such as teachers, students, community leaders): n = 3/79 (4%);
- more than one type of data collectors was present in 8 systems (10%).

Out of the 63 systems involving locally recruited surveillance cadres, 18 systems (29%) provided information on their selection processes. Selections were made either by community members (n = 12/18, 67%), local healthcare staff (n = 4/18, 22%), or by both community members and healthcare staff (n = 2/18, 11%). Information on the selection criteria used was available for 13 systems (21%), out of which two did not have formal selection criteria. Literacy was the most commonly used selection criterion (n = 10/11, 91%), which encompassed the ability to read and write (n = 7) and to hold at least a secondary level education (n = 3). Other frequently employed selection criteria included a motivation to work for the community (n = 6/11, 55%) and being someone respected in the community (n = 4/11, 36%).

Information on the use of incentives for data collectors to perform their duties was available for 21 systems (33%). In 16 systems (76%), data collection was performed on a voluntary basis. In 11 systems, the form of incentives given included monetary incentives (n = 4/11, 37%); material incentives (n = 3/11, 27%); service incentives (n = 1/11, 9%); both material and service incentives (n = 2/11, 18%); and both monetary and service incentives (n = 1/11, 9%).

Information on the training received by data collectors was provided for 18 systems (locally recruited surveillance cadres, n = 17; specific group of community members, n = 1). The training duration was: less than a week (n = 11/18, 61%), from one week to one month (n = 2/18, 11%), more than a month long (n = 5/18, 28%).

#### Data collection method

Three data collection methods were used in the CBS systems:

- Active data collection (n = 21/50, 42%): locally recruited surveillance cadres proactively searched for diseases or events under surveillance by making home visits (n = 21/21, 100%) and actively meeting and talking to the community members (n = 5/21, 24%).
- Passive data collection (n = 11/50, 22%): surveillance actors collected information when a sick person visited them for diagnosis or treatment (n = 8/11, 73%); or when they received information on the occurrence of an event under surveillance (n = 3/11, 27%).
- Self-collection and reporting (n = 13/50, 26%): general community members collected information about themselves or their families and reported it, this was primarily used for triatomine bugs surveillance (n = 8/13, 62%) and for surveillance of influenza like illness (n = 4/13, 31%). In five systems (10%), several data collection methods were applied.

#### Data reporting method

In most systems, surveillance actors visited a supervisor or vice-versa to submit or collect reports (n = 23/37, 62%). In 35% of the systems, reporting was done through telecommunication (n = 13/37), using a combination of phone calls (n = 6), mobile phone applications or SMS (n = 5), websites (n = 4), fax (n = 1), or wireless radio (n = 1). In one system, reporting involved both making visits and making phone calls [41]. All systems reporting via telecommunication were implemented in the last 15 years (n = 11, two systems had missing information for their start year). All systems started after 2010 reported via telecommunication (n = 4, three systems had missing information for the reporting method). The four CBS systems using websites to report were implemented in high-income countries for self-reporting of influenza like illness.

Most systems reported data in a routine manner using predetermined schedule (n = 37/60, 62%): weekly (n = 12), monthly (n = 18), less than monthly (n = 11), combination of several frequencies (n = 6). In 30% of the systems (n = 18/60), data was reported on an ad hoc manner; whilst 8% of the systems (n = 5/60) reported data in both an ad hoc and routine manner. In 59% of the systems with an early detection purpose, reporting was done in an ad hoc manner (n = 16/27), while in 90% of the systems with a monitoring purpose, reporting was done in a routinely manner (n = 28/31). Reporting was commonly done to the local level (n = 46/54, 86%), and the most common recipient was a health authority (n = 41/57, 72%).

#### Performance indicators

Estimates of the sensitivity (i.e. capacity of the system to detect the events under surveillance) or of the positive predictive value (i.e. capacity of the system to correctly detect the events under surveillance) were available for seven CBS systems (see Table 3). The completeness of data reporting was provided for ten CBS systems (see Table 4).

**Table 3:** Estimates of the sensitivity and positive predictive value of CBS systems.

Country [ref]	Methodology	Sensitivity calculation	Positive predictive value calculation	Period of interest	Scope	Sensitivity estimate	Positive predictive value estimate
Benin [42]	Cross-sectional household survey: in 2011 surveyors visited all households covered by the CBS system to collect the same information as collected by the CBS system in 2010.	Not specified	/	2010	Maternal death	95%	/
					Infant death	47%	
					Under 5 death	48%	
Cambodia [4]	Cross-sectional household survey: surveyors visited households (in 3 out of 7 areas implementing CBS) to collect cases of diseases (preceding month) and vital events (preceding year), using the same case definitions as used by the CBS system. The survey was conducted once. For measles: outbreak investigation data.	(No. of cases/events identified both by the CBS system and the household survey) / (No. of cases or events identified by the household survey)	(No. of cases or events identified both by the CBS system and household survey) / (No. of cases identified by the CBS system)	One year (2000–2001)	Measles	93% (n = 86/92)	90% (n = 86/96)
					Birth	82% (n = 28/34)	100% (n = 28/28)
				One month (2001)	Severe diarrhoea	82% (n = 10/12)	82% (n = 10/12)
					Chronic cough	75% (n = 55/73)	89% (n = 55/62)
					Malaria	65% (n = 57/88)	88% (n = 57/65)
Ethiopia [43]	Cross-sectional survey: in randomly selected villages implementing the CBS system, the blood of suspect malaria cases identified by CBS actors were tested to confirm malaria.	/	(No. of malaria cases confirmed with blood test) / (No. of suspected malaria cases identified by the CBS system)	1995–1996	Malaria	/	93% (n = 1453/1562)
Nigeria [44,45]	Confirmatory follow-up visits by an investigator in the villages reported having new cases as well as villages reported having zero cases.	(No. of villages reported confirmed as having cases through the follow-up visit) / (Total No. of villages with verified cases of guinea worm)	(No. of villages reported confirmed as having cases through the follow-up visit) / (Total No. of villages with cases reported by the CBS system).	6 months (1990–1991)	Guinea-worm disease	79% (n = 50/63)	93% (n = 50/54)
Sierra Leone [46]	Suspected cases were confirmed by laboratory diagnostic test	(No. of confirmed cases detected by the CBS system) / (Total No. of confirmed cases identified in the area).	(No. of confirmed cases detected by the CBS system) / (Total No. of cased detected by CBS system (suspected, probable and confirmed)).	7 months (2015)	Ebola virus disease	30% (n = 16/53)	6% (n = 16/287)
Sweden [47]	One week recall survey: a sample of participants in the CBS system was sent a questionnaire to collect the occurrence of influenza like illness in the previous week. Each year each participant went through two-three validation surveys.	/	(No. of participants who reported having influenza like illness in both the CBS system and one-week recall survey) / (No. of participants who reported having influenza like illness in the CBS system)	Two 8-week period (in 2008 and 2009)	Influenza like illness	/	2008: 79% (n = 73/92); 2009: 88% (n = 70/80)
Tanzania [48]	Cross-sectional survey: investigators visited and searched for mosquito larvae habitat in randomly selected housing clusters (consisting of 20–100 houses) covered by CBS system.	(No. of mosquito larvae habitat identified by the CBS system in the areas covered by cross-sectional survey) / (No. of mosquito larvae habitat reported by investigator during the cross-sectional survey)	/	8 months (2007–2008)	Mosquito larvae habitat	66.2% (n = 1963/2965)	/

**Table 4:** Completeness of data reporting for CBS systems.

Country [ref]	Scope	Reporting rate calculation	Period of interest	Reporting frequency	Completeness of data reporting
**Ethiopia** [49]	Birth, Death	(No. of catchment areas submitting reports per month) / (Total No. of catchment areas (n = 183))	2012–2013	Monthly	95% on average
**Ethiopia** [43]	Malaria	(No. of surveillance actors submitting reports per month/week) / (Total No. of surveillance actors (about 2500))	1994–1998	Monthly	90% on average
				Weekly	60% on average
**Ghana** [50]	Acute flaccid paralysis, Meningitis, Measles, Neonatal tetanus, Guinea-worm disease, Buruli Ulcer, Birth, Death, Maternal death, Infant death, Unusual events	(Total No. of submitted reports) / (Total No. of expected reports (n = NA))	1999	Monthly	74% overall (range: 53%’94% for different districts)
**India** [5]	Diarrhoea, Malaria, Measles, Dengue, Meningitis, Acute respiratory illness, Tuberculosis, Acute flaccid paralysis, Unusual symptoms, Birth, Death	(Total No. of surveillance actors submitting reports) / (Total number of expected reports (n = 48))	Six weeks (2005)	Weekly	91.6% overall by women self-help groups; 66.6% overall by members of another village group.
**Laos** [51]	Malaria, Birth, Death	Village health volunteers interview (n = 137)	Three previous months (2014)	Monthly	12.4% stated they reported every month during the 3 previous months (n = 17/137); 27% made reports “some months” (n = 37/137); 60.6% indicated never sending reports (n = 83/137).
**Mali** [49]	Birth, Death	(No. of catchment areas submitting reports per month) / (Total No. of catchment areas (n = 78))	2012–2013	Monthly	100% on average
**Malawi** [49]	Birth, Death	(No. of catchment areas submitting reports per month) / (Total No. of catchment areas (n = 160))	2010–2013	Monthly	95% on average
**Nigeria** [45]	Guinea-worm disease	(No. of reports received per week) / (Expected number of reports per week (n = 164))	16 weeks (1990)	Weekly	84% on average
**Sierra Leone** [52]	Ebola virus disease	(No. of surveillance actors submitting reports per month) / (Total No. of surveillance actors (n = 7142))	Six months (2015)	Weekly	82% on average (range: 38%’92% for different months)
**South Sudan** [53]	Acute Flaccid Paralysis	Not specified	2005	Daily, Weekly	40.5%: average reporting rate in 2005 for each State.

### Supplemental study: Usage of the term “community-based surveillance” in the literature

Out of the 1494 unique search results from the scoping review, 232 documents used the term “community-based surveillance” in their title or abstract (full results in S2 Text). Description of the approach termed as “community-based surveillance”, including the source of data, was available for 177 documents.

Around one third of these documents used the term “community-based surveillance” to describe the approach we defined as CBS, where data was collected from the community by community members for public health surveillance purposes (31%, n = 54/177). All of these documents, except two, were included in our CBS scoping review (out of the two excluded documents, for one [147] there was collection but no reporting of information for public health surveillance, and for the other [148] CBS was discussed as one of the possible strategies for control of Buruli ulcer, without providing any specifics).

The second most frequent use of the term “community-based surveillance” in the literature was to denote a research design where information was collected from the community by surveyors or healthcare facility staff (28%, n = 50/177) for research purposes.

The third most frequent use of the term “community-based surveillance” met none of the criteria that we used in the scoping review to describe a CBS system (22%, n = 39/177). They generally described a specific research study where surveyors collected data on a sample of enrolled patients at healthcare facilities.

The other approaches termed as “community-based surveillance” were: community members collecting information from the community for research purposes (11%, n = 20); non-community members collecting information from the community for public health surveillance purposes (5%, n = 9); surveyors collecting information from healthcare facility patients for public health surveillance purposes (3%, n = 5).

## Discussion

### Summary of evidence

This scoping review retrieved 134 documents mentioning the approach of involving community members in identifying and reporting health events occurring in their community for public health surveillance. As many as one third of the documents did not use any term to characterize CBS, and amongst others, 44 unique terms were used. Only 10 documents provided a definition for CBS, showing a similar display of the lack of clarity surrounding CBS.

Seven major guidance documents on CBS were identified [9,12–14,24,27,28], including three guides solely focused on CBS [9,12,14]. Guidance and recommendations on CBS practices were identified in sixteen additional documents. Description of the specific activities required for CBS implementation and operations were scattered across several documents. Their consolidation into a single process, with clear expectations on the roles and responsibilities of the different actors involved, would be highly beneficial to facilitate the set up and operation of a CBS system. A similar case is also noted for recommendations related to the best modalities for the selection, training, and incentivisation of locally recruited community members for CBS.

This review identified 79 unique examples of CBS systems implemented since 1958 across 42 countries. They were mostly implemented in low and lower-middle income countries (79%), and appeared to be fragmented (81% covering a limited geographical area and 70% solely implemented in a rural setting), vertical (67% with a single scope of interest), and of limited duration (median duration of operation: 6 years for ongoing systems and 2 years for ended systems). This highlights the lack of scale up of pilot programs, and the lack of integration of CBS into the overall national public health surveillance system. CBS implementation was mainly performed in rural settings and the best approaches to implement it in urban settings were still to be defined [50].

Only 72% of the systems provided information on their purpose: 45% were implemented solely to monitor the health status of a population, 30% solely to early detect and respond to public health events, and 25% for both purposes. Eighty percent of the systems recruited community members as volunteers or paid workers to collect and report data, the others relied on general community members or a specific group in the community.

A surge in the use of telecommunication for CBS reporting has taken place in the last fifteen years, which is linked with the dramatic surge of phone connectivity in most countries. The use of telecommunication creates an opportunity to enhance completeness and timeliness of reporting [51,54,55] and to improve data management. However, the specific challenges generated by the use of digital tools for public health surveillance, such as their cost and sustainability, cannot be ignored [149].

Only a fraction of the documents provided evaluation results of the implemented systems. Estimates of sensitivity and positive predictive value were available for seven systems, and results of completeness of data reporting for ten. However, these estimates were computed in an inconsistent manner, and usually for a short time duration, making it difficult to generalize or compare findings. Minimum requirements and sound methodology to evaluate CBS systems and disseminate evaluation results are thus urgently needed.

### Limitations

The main limitation of included documents was the inconsistent manner in which information on CBS systems was available, with a lot of missing information for several aspects of the systems. One explanation is our broad inclusion criteria which included documents that did not have a main focus on the description of a CBS system, but merely mentioned its existence. We tried as much as possible to correct this limitation by consolidating all available information for each specific system from several documents.

For this scoping review, we strove to apply best standards with double-blind screening and data collection, discrepancies being solved through consensus. We tried to be as sensitive as possible using tailored search algorithms to each bibliographic database, specific terms to search the worldwide web, screening the references of each included document, removing any time limits, and looking at publications in four languages (English, French, Portuguese and Spanish). Exclusion of papers based on language may have missed some CBS implementations, especially in Asia. There is also a risk that a publication bias may have favoured externally supported CBS implementations, the existence of such a bias and its magnitude are yet to be studied.

The major challenge we faced for this scoping review was to decide what should be considered as “community-based surveillance”. Indeed, lack of prior consensus in the term and definition for CBS mandated that we define in advance what should be encompassed in the CBS concept. We decided as minimum requirements that community members be both the source of information and the actors collecting it, and that this information be used for public health surveillance purposes. In addition, we had to define what we considered as a community. For the sake of simplicity, we defined a community as people living in a defined geographical area, excluding healthcare facilities from the community level. The rationale behind the exclusion of healthcare facilities was to avoid healthcare facility-based surveillance systems that are already well-known and broadly used for public health surveillance. With our inclusion criteria we considered any document presenting both concepts of community and public health surveillance. This may explain why a third of the included documents didn’t use any term to denote CBS. To ensure the validity of our inclusion criteria we conducted a supplemental study on the approaches termed as “community-based surveillance” in the literature (see S2 Text). Only 22% of the documents with mention of the term “community-based surveillance” in their title or abstract were included in our scoping review. Indeed, the sole purpose of 61% of these approaches termed as “community-based surveillance” was research. For the remaining approaches termed as “community-based surveillance”, and aimed at public health surveillance, 79% fulfilled our inclusion criteria of community members being both the source of information and the actors collecting it.

To our knowledge, and the best of our search efforts, this is the first scoping review on CBS to date. In 2002, Oum has conducted a previous narrative review on CBS as part of his Doctorate in Public Health [150], documents of interest from his review were included in ours.

### Conclusions

This scoping review, through the mapping of practices, guidance and recommendations on CBS, provides the foundational work to standardize and improve the involvement of community members in identifying and reporting health events occurring in their community for public health surveillance. As such, in June 2018, the results of this scoping review were presented to international experts convened by the World Health Organization [151]. They used these results and their experience to reach a consensus on the term “community-based surveillance” and its definition: “Community-based surveillance is the systematic detection and reporting of events of public health significance within a community by community members” [151]. They also agreed on a list of good practices and challenges for CBS and provided a list of priority activities to be conducted to further promote and support CBS implementation. The top three proposed activities were: develop and compile case studies of existing CBS, consolidate existing guidance and fulfil existing knowledge gaps in global CBS guidelines, and create a CBS community of practice with a shared repository of available material [151].

It was no surprise that a large majority of the CBS systems identified in this scoping review were implemented in low and lower-middle income countries. Healthcare facility-based surveillance systems face numerous challenges in these countries [152,153], including: healthcare access; communication with hard to reach areas; lack of human, logistic and financial resources; lack of coordination between multiple surveillance systems; lack of use of data for response. The burden put by health information systems on healthcare facility staff is often overwhelming [154–156]. CBS can appear as an opportunity to tackle some of these challenges. Yet, these challenges should also be stark reminders of the need to carefully craft CBS systems to their specific setting, so that their contribution to the public health surveillance system is not hindered by the creation of an additional vertical system, or by adding undue burden on selected community members [157]. Further research is needed to do so. A first step could indeed be to consolidate available guidance and recommendations, and develop standardized protocols and indicators to evaluate the effectiveness and integration of existing CBS systems into the overall health information system.

## Supporting information

S1 Table Search strategies and results. (PDF)

S2 Table CBS review evidence tables. (PDF)

S1 Text Summary of available guidance and recommendations for community-based surveillance. (PDF)

S2 Text Supplemental study on the usage of the term “community-based surveillance” in the literature. (PDF)

S1 File PRISMA-SrC checklist. (PDF)
