# Supplementary material for: Community-based surveillance: A scoping review
Source: PLoS One. 2019 Apr 12;14(4):e0215278. doi: 10.1371/journal.pone.0215278 (PMC6461245; doi:10.1371/journal.pone.0215278)
Supplement: S1 Table — (PDF) [file pone.0215278.s001.pdf]

Supporting table 1 – Search strategies and results

Database	Search date	Search request	No. of results	Unique results
Medline	28/03/2017	(("sentinel surveillance"[MeSH Terms] OR "population surveillance"[MeSH Terms] OR "public health surveillance"[MeSH Terms] OR surveillance [Title/Abstract] OR "public health surveillance") AND ("Community-Based Participatory Research"[MeSH Terms] OR "Community-Institutional Relations"[MeSH Terms] OR "Community Health Workers"[MeSH Terms] OR "volunteers" [MeSH Terms]))<br>OR "community-based surveillance"[TIAB] OR "participatory surveillance"[TIAB] OR "household surveillance"[Title/Abstract] OR "community based sentinel surveillance" [TIAB] OR "community based health reporting" [TIAB]	492	492
EMBASE	05/05/2017	(surveillance NEAR/3 ('community level' OR 'community based' OR 'community health workers' OR 'lay health' OR 'frontline health' OR 'community directed')):ti,ab<br>OR<br>(surveillance NEAR/9 community NEAR/9 volunteer*):ti,ab,de<br>OR<br>(surveillance NEAR/9 community NEAR/9 'worker'):ti,ab,de<br>OR<br>'health auxiliary'/exp OR 'voluntary worker'/exp OR 'community participation':ti,ab OR 'household based':ti,ab OR 'community reporting':ti,ab OR 'community based':ti,ab OR (('community health' OR 'lay health' OR 'community based' OR 'frontline health') NEAR/2 (worker* OR healer*)):ti,ab<br>AND surveillance:t	626	626
Global Index Medicus	19/04/2017	(tw:("Community-based surveillance")) OR (tw:("Community event based surveillance")) OR (tw:("Community based active surveillance")) OR (tw:(" Community based health reporting")) OR (tw:(" Community based vital events reporting")) OR (tw:(" Community based disease reporting")) OR (tw:("Community based reporting")) OR (tw:("Community based household surveillance")) OR (tw:("Community based monitoring")) OR (tw:("Community based disease monitoring")) OR (tw:("Community participatory surveillance"))	224	272
	21/04/2017	((mh:("community health workers")) AND ((mh:("Population surveillance")) OR (mh:("Public health surveillance")) OR (mh:("Sentinel surveillance")) OR (mh:("Epidemiological surveillance")) OR (mh:("Epidemiological monitoring")))) AND (instance:"ghl")	61	
Popline	20/04/2017	(((Title:"community\-based" OR Title:"community based" OR Title:"community participatory"))) AND (((surveillance)))	70	
		((("community\-based" OR "community based" OR "community participatory"))) AND (((Title:monitoring OR Title:surveillance)))	92	
		(((Title:"community based" OR Title:"community\-based" OR Title:"community participatory"))) AND (((Keyword:DATA REPORTING OR Keyword:DATA COLLECTION)))	122	491
		(((Keyword:COMMUNITY PARTICIPATION OR Keyword:COMMUNITY WORKERS))) AND (((Title:surveillance OR Title:monitoring)))	80	
	21/04/2017	(((Keyword:COMMUNITY WORKERS) AND (Keyword:DATA REPORTING OR Keyword:DATA COLLECTION)))	205	
Cochrane library	16/04/2017	#1 MeSH descriptor: [Population Surveillance] explode all trees<br>#2 MeSH descriptor: [Disease Notification] explode all trees<br>#3 MeSH descriptor: [Epidemiological Monitoring] explode all trees<br>#4 "household surveillance":ti,ab,kw (Word variations have been searched)<br>#5 "health information system":ti,ab,kw (Word variations have been searched)<br>#6 "sentinel surveillance":ti,ab,kw (Word variations have been searched)<br>#7 MeSH descriptor: [Community Networks] explode all trees<br>#8 MeSH descriptor: [Consumer Participation] explode all trees<br>#9 MeSH descriptor: [Community Health Workers] explode all trees<br>#10 MeSH descriptor: [Volunteers] explode all trees<br>#11 "community-based surveillance":ti,ab,kw (Word variations have been searched)<br>#12 "community based monitoring":ti,ab,kw (Word variations have been searched)<br>#13 "community based reporting":ti,ab,kw (Word variations have been searched)<br>#14: #1 or #2 or #3 or #4 or #5 or #6<br>#15: #7 or #8 or #9 or #10<br>#16: #14 and #15<br>#17: #11 or #12 or #13 or #16	31	31
European library	20/04/2017	"advanced((TITLE,community based)AND(TITLE,surveillance))"	26	
		"advanced((TITLE,community based)AND(TITLE,reporting))"	2	31
		"community-based surveillance"	36	

Database	Search date	Search request	No. of results	Unique results
Africabib	20/04/2017	ab=community surveillance	14	15
		su=surveillance; ti=Community	1	
		ab=Surveillance; ti=Community	3	
		ab=Community; ti=surveillance	2	
Google	17/12/2015	Community-based surveillance		18
	05/01/2016	Community event based surveillance filetype:pdf		
	06/01/2016	Community based early warning filetype:pdf		
	06/01/2016	Community based disease surveillance filetype:pdf		
