# Supplementary material for: Community-based surveillance: A scoping review
Source: PLoS One. 2019 Apr 12;14(4):e0215278. doi: 10.1371/journal.pone.0215278 (PMC6461245; doi:10.1371/journal.pone.0215278)
Supplement: S2 Table — (PDF) [file pone.0215278.s002.pdf]

Supporting table 2 – CBS review evidence table

List of included documents

Doc Id	Reference	Type of document	Term used for CBS
1	Dyal Chand A, Khale M. A community based surveillance system for perinatal and neonatal care. *Indian Pediatr* 1989; **26**: 1115–21.	Scientific paper	Community-based surveillance and monitoring
2	Asres M, Bisrat F, Kebede Y, Asegedew B, Getachew B, Fantahun M. Knowledge and practice of frontline health workers (Health Extension Workers and Community Volunteer Surveillance Focal Persons) towards acute flaccid paralysis (AFP) case detection and reporting in pastoralist and semi-pastoralist areas of Ethiopia. *Ethiop Med J* 2013; **51**: 51–7.	Scientific paper	Community-based surveillance
3	Bisimwa G, Mambo T, Mitangala P, Schirvel C, Porignon D, Dramaix M, et al. Nutritional monitoring of preschool-age children by community volunteers during armed conflict in the Democratic Republic of the Congo. *Food Nutr Bull* 2009; **30**: 120–7.	Scientific paper	No term
4	Deepa TM, Venkata Rao E, Patil RR, Samuel R. Operational feasibility of establishing community reporting systems. *Natl Med J India* 2008; **21**: 166–70.	Scientific paper	Community reporting system
5	Dil Y, Strachan D, Cairncross S, Korkor AS, Hill Z. Motivations and challenges of community-based surveillance volunteers in the northern region of Ghana. *J Community Health* 2012; **37**: 1192–8.	Scientific paper	Community-based surveillance
6	Hashimoto K, Zúniga C, Nakamura J, Hanada K. Integrating an infectious disease programme into the primary health care service: a retrospective analysis of Chagas disease community-based surveillance in Honduras. *BMC Health Serv Res* 2015. DOI 10.1186/s12913-015-0785-4	Scientific paper	Community-based vector surveillance<br>Community-based surveillance
7	Kilonzo A, Kouletio M, Whitehead SJ, Curtis KM, McCarthy BJ. Improving surveillance for maternal and perinatal health in 2 districts of rural Tanzania. *Am J Public Health* 2001; **91**: 1636–40.	Scientific paper	Community-based surveillance
8	Kok MC, Muula AS. Motivation and job satisfaction of health surveillance assistants in Mwanza, Malawi: an explorative study. *Malawi Med J* 2013; **25**: 5–11.	Scientific paper	No term
9	Dinku B, Kumie A, Bisrat F. Linking community volunteer surveillance focal persons with health extension workers on polio surveillance. *Ethiop Med J* 2013; **51**: 71–6.	Scientific paper	Community-based acute flaccid paralysis-Measles and Neonatal Tetanus (NT) surveillance
10	Okanurak K, Sornmani S, Chitprarop U. The role of folk healers in the malaria volunteer program in Thailand. *Southeast Asian J Trop Med Public Health* 1991; **22**: 57–64.	Scientific paper	No term
11	Wójcik OP, Brownstein JS, Chunara R, Johansson MA. Public health for the people: participatory infectious disease surveillance in the digital age. *Emerging themes in epidemiology* 2014; **11**: 7.	Scientific paper	Internet-based participatory surveillance
12	Ngirabega JDD, Hakizimana C, Wendy L, Munyanshongore C, Donnen P, Dramaix-Wilmet M. Reliability of anthropometric measurements performed by community nutrition workers in a community-based pediatric growth-monitoring program in rural Rwanda. *Rev Epidemiol Sante Publique* 2010; **58**: 409–14.	Scientific paper	No term
13	Bose A, Sandal Sejbaek C, Suganthy P, Raghava V, Alex R, Muliyil J, et al. Self-harm and self-poisoning in southern India: choice of poisoning agents and treatment. *Trop Med Int Health* 2009; **14**: 761–5.	Scientific paper	Community-based surveillance
14	Afele M. Volunteers vital for counting births and deaths in Ghana. *Bulletin of the World Health Organization* 2011; **89**: 322–3.	Scientific paper	Community-based surveillance
15	Hashimoto K, Yoshioka K. Review: surveillance of Chagas disease. *Adv Parasitol* 2012; **79**: 375–428.	Book chapter	Community-based surveillance
16	Integrated disease surveillance and response in the African Region: a guide for establishing community based surveillance. Brazzaville: World Health Organization Regional Office for Africa, 2014.	Guidance document	Community-based surveillance
17	Evaluation of the functionality and effectiveness of community event based surveillance in Sierra Leone. UKaid and Ebola Response Consortium, 2015.	Report	Community event-based surveillance
18	Integrated Diseases Surveillance and Response in the African Region. Community-based Surveillance (CBS) Training Manual. Brazzaville: World Health Organization Regional Office for Africa, 2015.	Training manual	Community-based surveillance

Doc Id	Reference	Type of document	Term used for CBS
19	Lado M, Mackoy S, Steve B, Rumunu J. Evaluation of community-based surveillance for Guinea worm, South Sudan, 2006. *SSMJ* 2012; **5**: 72–4.	Scientific paper	Community-based surveillance
20	Quarterly report "Community event-based surveillance (CEBS) in Sierra Leone." International Rescue Committee Sierra Leone Program; 2015.	Report	Community event-based surveillance
21	Standard Operating Procedure for community event-based surveillance for Ebola virus disease in Sierra Leone. New York: International Rescue Committee, 2015.	SOPs	Community event-based surveillance
22	Brieger WR, Kendall C. The Yoruba farm market as a communication channel in guinea worm disease surveillance. *Soc Sci Med* 1996; **42**: 233–43.	Scientific paper	Community-based surveillance<br>Market-based surveillance
23	Hashimoto K, Alvarez H, Nakagawa J, Juarez J, Monroy C, Cordón-Rosales C, et al. Vector control intervention towards interruption of transmission of Chagas disease by Rhodnius prolixus, main vector in Guatemala. *Mem Inst Oswaldo Cruz* 2012; **107**: 877–87.	Scientific paper	Community-based surveillance
24	Hii JL, Chee KC, Vun YS, Awang J, Chin KH, Kan SK. Sustainability of a successful malaria surveillance and treatment program in a Runggus community in Sabah, east Malaysia. *Southeast Asian J Trop Med Public Health* 1996; **27**: 512–21.	Scientific paper	Community-based malaria surveillance<br>Community surveillance
25	Kyei-Faried S, Appiah-Denkyira E, Brenya D, Akuamoa-Boateng A, Visser L. The Role of Community-Based Surveillance in Health Outcomes Measurement. *Ghana Med J* 2006; **40**: 26–30.	Scientific paper	Community-based surveillance
26	Lapau B. The role of village malaria workers as village surveillance agents in Bekasi Regency, Indonesia. S*outheast Asian J Trop Med Public Health* 1983; **14**: 12–7.	Scientific paper	No term
27	Nonaka D, Pongvongsa T, Nishimoto F, Nansounthavong P, Hongwei J, Vongsouvanh A, et al. Successful mobile phone network-based approach to integration of the health care system in rural Laos: strengthening lay health worker performance. *Rural Remote Health* 2014; **14**: 2588.	Scientific paper	Community-based surveillance
28	Nsona H, Mtimuni A, Daelmans B, Callaghan-Koru JA, Gilroy K, Mgalula L, et al. Scaling up integrated community case management of childhood illness: update from Malawi. *Am J Trop Med Hyg* 2012; **87**: 54–60.	Scientific paper	No term
29	Paolotti D, Carnahan A, Colizza V, Eames K, Edmunds J, Gomes G, et al. Web-based participatory surveillance of infectious diseases: the Influenzanet participatory surveillance experience. *Clin Microbiol Infect* 2014; **20**: 17–21.	Scientific paper	No term
30	Purdin S, Spiegel P, Mack KP, Millen J. Surveillance beyond camp settings in humanitarian emergencies: findings from the Humanitarian Health Information Management Working Group. *Prehosp Disaster Med* 2009; 24: s202-205.	Scientific paper	Community-based surveillance<br>Community-based information collection
31	Ramsey K, Hingora A, Kante M, Jackson E, Exavery A, Pemba S, et al. The Tanzania Connect Project: a cluster-randomized trial of the child survival impact of adding paid community health workers to an existing facility-focused health system. *BMC Health Serv Res* 2013; **13**: S6.	Scientific paper	No term
32	Ruebush TK, Weller SC, Klein RE. Qualities of an ideal volunteer community malaria worker: a comparison of the opinions of community residents and national malaria service staff. *Soc Sci Med* 1994; **39**: 123–31.	Scientific paper	No term
33	Smith S, Deveridge A, Berman J, Negin J, Mwambene N, Chingaipe E, et al. Task-shifting and prioritization: a situational analysis examining the role and experiences of community health workers in Malawi. *Hum Resour Health* 2014; **12**: 24.	Scientific paper	No term
34	Waiswa P, Peterson SS, Namazzi G, Ekirapa EK, Naikoba S, Byaruhanga R, et al. The Uganda Newborn Study (UNEST): an effectiveness study on improving newborn health and survival in rural Uganda through a community-based intervention linked to health facilities - study protocol for a cluster randomized controlled trial. *Trials* 2012; **13**: 213.	Scientific paper	No term
35	Cerón A, Ortiz MR, Álvarez D, Palmer GH, Cordón-Rosales C. Local disease concepts relevant to the design of a community-based surveillance program for influenza in rural Guatemala. *Int J Equity Health* 2016; **15**: 69.	Scientific paper	Community-based surveillance
36	Brookes VJ, Kennedy E, Dhagapan P, Ward MP. Qualitative Research to Design Sustainable Community-Based Surveillance for Rabies in Northern Australia and Papua New Guinea. *Front Vet Sci* 2017; **4**: 19.	Scientific paper	Community-based surveillance
37	Chaki PP, Mlacha Y, Msellemu D, Muhili A, Malishee AD, Mtema ZJ, et al. An affordable, quality assured community-based system for high-resolution entomological surveillance of vector mosquitoes that reflects human malaria infection risk patterns. *Malaria J* 2012; **11**: 172.	Scientific paper	Community-based surveillance

Doc Id	Reference	Type of document	Term used for CBS
38	Abad-Franch F, Vega MC, Rolón MS, Santos WS, Rojas de Arias A. Community participation in Chagas disease vector surveillance: systematic review. *PLoS Negl Trop Dis* 2011; **5**: e1207.	Scientific paper	No term
39	Bowden S, Braker K, Checchi F, Wong S. Implementation and utilisation of community-based mortality surveillance: a case study from Chad. *Confl Health* 2012; **6**: 11.	Scientific paper	Community-based mortality surveillance
40	Chau PD. Evaluation of Disease Surveillance System to Detect Disease Outbreak in Cambodia. *J. Natl. Inst. Public Health* 2007; **56**: 412.	Scientific paper	Community-based surveillance
41	Crowe S, Hertz D, Maenner M, Ratnayake R, Baker P, Lash RR, et al. A plan for Community Event-Based Surveillance to Reduce Ebola Transmission — Sierra Leone, 2014–2015. *Morbidity and Mortality Weekly Report* 2015; **64**: 70–3.	Scientific paper	Community event-based surveillance
42	Maes EF, Zimicki S. An evaluation of community-based surveillance in the northern region of Ghana. UNICEF, 2000.	Report	Community-based surveillance
43	Okiror SO, Bisrat F, Lutukai M, Bhui BR. Community-based surveillance on polio eradication in the Horn of Africa. *African Health Monitor* 2015; **19**: 44–5.	Scientific paper	Community-based surveillance
44	Wendy Morotti, Briac V, Papowitz H. Community-Based Pandemic Preparedness. Multi-sectorial actions for safer, healthier and more resilient communities.	Report	Community-based early warning and surveillance
45	A Guide to Establishing Event-based Surveillance. Manila: World Health Organization Western Pacific Region, 2008.	Guidance document	No term
46	Abass KM, Werf TS van der, Phillips RO, Sarfo FS, Abotsi J, Mireku SO, et al. Buruli ulcer controlin a highly endemic district in Ghana: role of community-based surveillance volunteers. *Am J Trop Med Hyg* 2015; **92**: 115–7.	Scientific paper	No term
47	Anselmi M, Moreira J-M, Caicedo C, Guderian R, Tognoni G. Community participation eliminates yaws in Ecuador. *Trop Med Int Health* 2003; **8**: 634–8.	Scientific paper	Community-based surveillance
48	Bajardi P, Vespignani A, Funk S, Eames KT, Edmunds WJ, Turbelin C, et al. Determinants of follow-up participation in the Internet-based European influenza surveillance platform Influenzanet. *J Med Internet Res* 2014; **16**: e78.	Scientific paper	Internet-based community surveillance
49	Cairncross S, Braide EI, Bugri SZ. Community participation in the eradication of guinea worm disease. *Acta Trop* 1996; **61**: 121–36.	Scientific paper	Village-basedsurveillance<br>Community-based surveillance
50	Choi Y, El Arifeen S, Mannan I, Rahman SM, Bari S, Darmstadt GL, et al. Can mothers recognize neonatal illness correctly? Comparison of maternal report and assessment by community health workers in rural Bangladesh. *Trop Med Int Health* 2010; **15**: 743–53.	Scientific paper	No term
51	Cox J, Dy Soley L, Bunkea T, Sovannaroth S, Soy Ty K, Ngak S, et al. Evaluation of community-based systems for the surveillance of day three-positive Plasmodium falciparum cases in Western Cambodia. *Malar J* 2014; **13**: 282.	Scientific paper	Community-based surveillance
52	Cwik MF, Barlow A, Goklish N, Larzelere-Hinton F, Tingey L, Craig M, et al. Community-Based Surveillance and Case Management for Suicide Prevention: An American Indian Tribally Initiated System. *Am J Public Health* 2014; **104**: e18–23.	Scientific paper	Community-based surveillance
53	Darmstadt G, El Arifeen S, Choi Y, Bari S, Rahman S, Mannan I, et al. Household surveillance of severe neonatal illness by community health workers in Mirzapur, Bangladesh: coverage and compliance with referral. *Health policy and planning* 2010; **25**: 112–24.	Scientific paper	No term
54	Goutard FL, Binot A, Duboz R, Rasamoelina-Andriamanivo H, Pedrono M, Holl D, et al. How to reach the poor? Surveillance in low-income countries, lessons from experiences in Cambodia and Madagascar. *Prev Vet Med* 2015; **120**: 12–26.	Scientific paper	No term
55	Imourou BCA, Perini P, Sohoundé L, Ahanhanzo C. Surveillance en communauté des décès maternels et infantojuvéniles dans le district sanitaire de Tanguiéta (Bénin) de 2006 à 2010. *Med Sante Trop* 2013; **23**: 332–6.	Scientific paper	No term
56	Kaneko A. A community-directed strategy for sustainable malaria elimination on islands: short-term MDA integrated with ITNs and robust surveillance. *Acta Trop* 2010; **114**: 177–83.	Scientific paper	Community-based surveillance
57	Mangklasriri R, Pichaipat V, Varavithya W. Effectiveness of diarrhoeal diseases surveillance by village health volunteer. *J Med Assoc Thai* 1986; **69**: 91–3.	Scientific paper	No term
58	Moshabela M, Sene M, Nanne I, Tankoano Y, Schaefer J, Niang O, et al. Early detection of maternal deaths in Senegal through household-based death notification integrating verbal and social autopsy: a community-level case study. *BMC Health Serv Res* 2015; **15**: 16.	Scientific paper	Community-level surveillance<br>Community-based surveillance

Doc Id	Reference	Type of document	Term used for CBS
59	N'Goran AA, Ilunga N, Coldiron ME, Grais RF, Porten K. Community-based measles mortality surveillance in two districts of Katanga Province, Democratic Republic of Congo. *BMC Res Notes* 2013; **6**: 537.	Scientific paper	Community-based measles mortality surveillance
60	Oum S, Chandramohan D, Cairncross S. Community-based surveillance: a pilot study from rural Cambodia. *Trop Med Int Health* 2005; **10**: 689–97.	Scientific paper	Community-based surveillance
61	Pongvongsa T, Nonaka D, Kobayashi J, Mizoue T, Phongmany P, Moji K. Determinants of monthly reporting by village health volunteers in a poor rural district of Lao PDR. *Southeast Asian J Trop Med Public Health* 2011; **42**: 1269–81.	Scientific paper	Community-based surveillance
62	Pyakurel R, Sharma N, Paudel D, Coghill A, Sinden L, Bost L, et al. Cause of Death in Women of Reproductive Age in Rural Nepal Obtained Through Community-Based Surveillance: Is Reducing Maternal Mortality the Right Priority for Women's Health Programs? *Health Care Women Int* 2015; **36**: 655–62.	Scientific paper	Community-based health information system
63	Ruebush TK, Godoy HA. Community participation in malaria surveillance and treatment. I. The Volunteer Collaborator Network of Guatemala. *Am J Trop Med Hyg* 1992; **46**: 248–60.	Scientific paper	No term
64	Ruebush TK, Zeissig R, Koplan JP, Klein RE, Godoy HA. Community participation in malaria surveillance and treatment. III. An evaluation of modifications in the Volunteer Collaborator Network of Guatemala. *Am J Trop Med Hyg* 1994; **50**: 85–98.	Scientific paper	No term
65	Sharma R, Ratnesh L, Karad AB, Kandpal H, Dhariwal AC, Ichhupujani RL. Communicable disease outbreak detection by using supplementary tools to conventional surveillance methods under Integrated Disease Surveillance Project (IDSP). *India. J Commun Dis* 2009; **41**: 149–59.	Scientific paper	Community-based surveillance
66	Assessment of the Community-Based Surveillance System in Ghana and its role in dracunculiasis eradication. *Wkly Epidemiol Rec* 2003; **78**: 321–3.	Scientific paper	Community-based surveillance
67	Joos O, Silva R, Amouzou A, Moulton LH, Perin J, Bryce J, et al. Evaluation of a mHealth Data Quality Intervention to Improve Documentation of Pregnancy Outcomes by Health Surveillance Assistants in Malawi: A Cluster Randomized Trial. *PLoS ONE* 2016; **11**: e014538.	Scientific paper	Community-based vital event documentation
68	Larsen DA, Chisha Z, Winters B, Mwanza M, Kamuliwo M, Mbwili C, et al. Malaria surveillance in low-transmission areas of Zambia using reactive case detection. *Malar J* 2015; **14**: 465.	Scientific paper	No term
69	Leal-Neto OB, Dimech GS, Libel M, Oliveira W, Ferreira JP. Digital disease detection and participatory surveillance: overview and perspectives for Brazil. *Rev Saude Publica* 2016; **50**: 17.	Scientific paper	No term
70	Meyers DJ, Ozonoff A, Baruwal A, Pande S, Harsha A, Sharma R, et al. Combining Healthcare-Based and Participatory Approaches to Surveillance: Trends in Diarrheal and Respiratory Conditions Collected by a Mobile Phone System by Community Health Workers in Rural Nepal. *PLoS ONE* 2016; **11**: e0152738.	Scientific paper	Community health worker based surveillance
71	Mitsunaga T, Hedt-Gauthier BL, Ngizwenayo E, Farmer DB, Gaju E, Drobac P, et al. Data for Program Management: An Accuracy Assessment of Data Collected in Household Registers by Community Health Workers in Southern Kayonza, Rwanda. *J Community Health* 2015; **40**: 625–32.	Scientific paper	No term
72	Pagliari, Claudia, and Santosh Vijaykumar. Digital Participatory Surveillance and the Zika Crisis: Opportunities and Caveats. *PLoS Neglected Tropical Diseases* 2016; **10**: e0004795	Scientific paper	Digital participatory surveillance
73	Pini A, Merk H, Carnahan A, Galanis I, VAN Straten E, Danis K, et al. High added value of a population-based participatory surveillance system for community acute gastrointestinal, respiratory and influenza-like illnesses in Sweden, 2013-2014 using the web. *Epidemiol Infect* 2017; **145**: 1193-202.	Scientific paper	No term
74	Ratnayake R, Crowe SJ, Jasperse J, Privette G, Stone E, Miller L, et al. Assessment of Community Event-Based Surveillance for Ebola Virus Disease, Sierra Leone, 2015. *Emerging Infect Dis* 2016; **22**: 1431–7.	Scientific paper	Community event-based surveillance
75	Silva R, Amouzou A, Munos M, Marsh A, Hazel E, Victora C, et al. Can Community Health Workers Report Accurately on Births and Deaths? Results of Field Assessments in Ethiopia, Malawi and Mali. *PLoS ONE* 2016; **11**: e0144662.	Scientific paper	Community-based vital events reporting
76	Chorlton R. Improving child survival and nutrition: the joint WHO/UNICEF Nutrition Support Programme in Iringa, Tanzania. Dar es Salaam: UNICEF, 1989.	Scientific paper	Community-based growth monitoring<br>Village-based monitoring,<br>Village-based nutritional status and death monitoring
77	Meyers DJ, Filkins M, Bangura AH, Sharma R, Baruwal A, Pande S, et al. Management challenges in mHealth: Failures of a mobile community health worker surveillance programme in rural Nepal. *BMJ Innov* 2017; **3**: 19–25	Scientific paper	No term

Doc Id	Reference	Type of document	Term used for CBS
78	Bugri DS. Community-based surveillance in Ghana. National surveillance unit, 2005.	Report	Community-based surveillance
79	Shayo E, Mboera LEG, MMbuji P, Rumisha SF, Senkoro KP, Mwami AJ. The role of community and traditional healers in communicable disease surveillance and management in Babati and Dodoma Districts, Tanzania. *Tanzania Health Research Bulletin* 2003; **5**: 48–55.	Scientific paper	Community-based disease surveillance
80	Chaki PP, Dongus S, Fillinger U, Kelly A, Killeen GF. Community-owned resource persons for malaria vector control: enabling factors and challenges in an operational programme in Dar es Salaam, United Republic of Tanzania. *Hum Resour Health* 2011; **9**: 21.	Scientific paper	Community-based surveillance
81	Joos O, Amouzou A, Silva R, Banda B, Park L, Bryce J, et al. Strengthening Community-Based Vital Events Reporting for Real-Time Monitoring of Under-Five Mortality: Lessons Learned from the Balaka and Salima Districts in Malawi. *PLoS One* 2016; **11**: e0145238.	Scientific paper	Community-based monitoring
82	Munos MK, Koffi AK, Sangho H, Traoré MG, Diakité M, Silva R. Strengthening Community Networks for Vital Event Reporting: Community-Based Reporting of Vital Events in Rural Mali. *PLoS One* 2015; **10**: e0132164.	Scientific paper	Community-based vital events reporting
83	Stone E, Miller L, Jasperse J, et al. Community Event-Based Surveillance for Ebola Virus Disease in Sierra Leone: Implementation of a National-Level System During a Crisis. *PLoS Curr*. 2016; **8**: ecurrents.outbreaks.d119c71125b5cce312b9700d744c56d8.	Scientific paper	Community event-based surveillance
84	Ahmed AEAM, Ahmed IAM. Nutrition surveillance in the Sudan: a community-based approach. *East Mediterranean Health Journal* 1996; **2**: 229–35.	Scientific paper	No term
85	Ebola and Marburg virus disease epidemics: preparedness, alert, control, and evaluation. Geneva: World Health Organization, 2014.	Scientific paper	Community-based surveillance
86	Role of village health volunteers in avian influenza surveillance in Thailand. New Delhi: World Health Organization Regional Office for South-East Asia, 2007.	Report	Community-based surveillance
87	Amaral J, Leite AJM, Cunha AJLA, Victora CG. Impact of IMCI health worker training on routinely collected child health indicators in Northeast Brazil. *Health Policy Plann* 2005; **20**: i42–8.	Scientific paper	Community health worker information system
88	Bellali H, Hchaichi A, Harizi C, Mrabet A, Chahed MK. Comparison between active surveillance and passive detection of zoonotic cutaneous leishmaniasis in endemic rural areas in Central Tunisia, 2009 to 2014. *Asian Pac J Trop Dis* 2015; **5**: 515–9.	Scientific paper	Community-based active ZCL surveillance
89	Fauveau V., Chakraborty J., Sarder A.M., Khan M.A., Koenig M.A. Measles among under- 9-month-olds in rural Bangladesh: Its significance for age at immunization. *BULL WHO* 1991; **69**: 67–72.	Scientific paper	Community-based measles surveillance
90	García-Zapata MT, Marsden PD. Chagas' disease: control and surveillance through use of insecticides and community participation in Mambaí, Goiás, Brazil. *Bull Pan Am Health Organ* 1993; **27**: 265–79.	Scientific paper	No term
91	Hashimoto K, Zúniga C, Romero E, Morales Z, Maguire JH. Determinants of health service responsiveness in community-based vector surveillance for Chagas disease in Guatemala, El Salvador, and Honduras. *PLoS Negl Trop Dis* 2015; 9: e0003974.	Scientific paper	Community-based vector surveillance
92	Kim SY, Rochat R, Rajaratnam A, Digirolamo A. Evaluating completeness of maternal mortality reporting in a rural health and social affairs unit in Vellore, India, 2004. *J Biosoc Sci* 2009; **41**: 195–205.	Scientific paper	Community-based death surveillance
93	Materia E, Mele A, Mehari W, Rosmini F, Stazi MA, Damen HM, et al. Estimation of early childhood mortality using preceding birth technique in a community-based setting. *Ann Ist Super Sanita* 1993; **29**: 465–7.	Scientific paper	No term
94	Ndiaye SM, Quick L, Sanda O, Niandou S. The value of community participation in disease surveillance: A case study from Niger. *Health Promot Int* 2003; **18**: 89–98.	Scientific paper	Community-based surveillance
95	Parente CC, Bezerra FSM, Parente PI, Dias-Neto RV, Xavier SCC, Ramos AN, et al. Community-based entomological surveillance reveals urban foci of chagas disease vectors in Sobral, State of Ceara, Northeastern Brazil. *PLoS ONE* 2017; **12**: e0170278.	Scientific paper	Community-based entomological surveillance
96	Handbook for community surveillance coordinators to support community participation in detection and prevention of polio and other diseases. Version 2. Washington, D.C: Academy for Educational Development, CHANGE Project, 2001.	Guidance document	Community surveillance
97	Amouzou A, Kidanu A, Taddesse N, Silva R, Hazel E, Bryce J, et al. Using Health Extension Workers for Monitoring Child Mortality in Real-Time: Validation against Household Survey Data in Rural Ethiopia. *PloS One* 2015; **10**: e0126909.	Scientific paper	Community monitoring of vital events
98	Barnett I, Yosellina, Sulistyo S, Befani B, KariSari K, Sharmin S, et al. Mixed-method impact evaluation of a mobile phone application for nutrition monitoring	Report	Community-based growth monitoring

Doc Id	Reference	Type of document	Term used for CBS
	in Indonesia. Institute for Development Studies, 2016.		
99	Edward A, Ernst P, Taylor C, Becker S, Mazive E. Examining the evidence of under-five mortality reduction in a community-based programme in Gaza, Mozambique. *Transactions of the Royal Society of Tropical Medicine and Hygiene* 2007; **101**: 814–22.	Scientific paper	Community-based vital registration<br>Community-based vital registration and disease surveillance
100	Freund P, Kalumba K. Information for health development. *World Health Forum* 1986; **7**: 185–90.	Scientific paper	Community-based information gathering
101	Gisore P, Shipala E, Otieno K, Rono B, Marete I, Tenge C, et al. Community based weighing of newborns and use of mobile phones by village elders in rural settings in Kenya: a decentralised approach to health care provision. *BMC Pregnancy and Childbirth* 2012; **12**: 15.	Scientific paper	No term
102	Hopkins D, Ruiz-Tiben E. Surveillance for dracunculiasis, 1981-1991. *Morbidity and Mortality Weekly Report: Surveillance Summaries* 1992; **41**: 1–13.	Scientific paper	No term
103	Immink M. Community-based food and nutrition surveillance as an instrument of socio-economic development in Central America: a point of view. *Food Nutr Bull* 1988; **10**: 13–5.	Scientific paper	Community-based food and nutrition surveillance
104	Jerome N, Ricci J. Food and nutrition surveillance: An international overview. *American Journal of Clinical Nutrition* 1997; **65**: 1198–1202.	Scientific paper	Community food and nutrition surveillance
105	Mahmood S, Ayub M. Accuracy of primary health care statistics reported by community based lady health workers in district Lahore. *Journal of the Pakistan Medical Association* 2010; **60**: 649–53.	Scientific paper	No term
106	BASICS II country report: Zambia. Arlington: Basic Support for Institutionalizing Child Survival Project (BASICS II) for the United States Agency for International Development, 2004.	Report	No term
107	Rosales A, Galindo J, Flores A. A community based surveillance system for maternal and early neonatal complications: the Intibuca case study. Baltimore: Catholic Relief Services, 2004.	Report	Community-based surveillance<br>Community-based health information system
108	Suva E. Gathering information for health. *World Health Forum* 1986; **7**: 340–4.	Scientific paper	No term
109	Curry D, Bisrat F, Coates E, Altman P. Reaching beyond the health post: Community-based surveillance for polio eradication. *Dev Pract* 2013; **23**: 69–78.	Scientific paper	Community-based surveillance
110	Dalton C, Durrheim D, Fejsa J, Francis L, Carlson S, d'Espaignet ET, et al. Flutracking: a weekly Australian community online survey of influenza-like illness in 2006, 2007 and 2008. *Commun Dis Intell Q Rep* 2009; **33**: 316–22.	Scientific paper	No term
111	Marquet RL, Bartelds AIM, van Noort SP, Koppeschaar CE, Paget J, Schellevis FG, et al. Internet-based monitoring of influenza-like illness (ILI) in the general population of the Netherlands during the 2003-2004 influenza season. *BMC Public Health* 2006; **6**: 242.	Scientific paper	No term
112	Noort SP va., Muehlen M, Rebelo de Andrade H, Koppeschaar C, Lima Lourenço JM, Gomes MGM. Gripenet: an internet-based system to monitor influenza-like illness uniformly across Europe. *Euro Surveill* 2007; **12**: E5-6.	Scientific paper	No term
113	Tilston NL, Eames KTD, Paolotti D, Ealden T, Edmunds WJ. Internet-based surveillance of Influenza-like-illness in the UK during the 2009 H1N1 influenza pandemic. *BMC Public Health* 2010; **10**: 650.	Scientific paper	No term
114	Informe: XIIa. Reunión Intergubernamental INCOSUR/Chagas. Santiago: Pan American Health Organization, 2003.	Report	No term
115	Villela MM, Souza JB, Mello VP, Azeredo BV de M, Dias JCP. Entomological surveillance for Chagas disease in the mid-western region of Minas Gerais State, Brazil, from 2000 to 2003. *Cad Saude Publica* 2005; **21**: 878–86.	Scientific paper	No term
116	Prata N, Gerdts C, Gessessew A. An innovative approach to measuring maternal mortality at the community level in low-resource settings using mid-level providers: a feasibility study in Tigray, Ethiopia. *Reprod Health Matters* 2012; **20**: 196–204.	Scientific paper	Community-based sentinel surveillance
117	Jaravaza VS, McCoy MC, Dando BC. Unified National Health Information System. Part II. The Village Health Worker Health Information System. *Cent Afr J Med* 1982; **28**: 57–65.	Scientific paper	Village health worker health information system
118	Valyasevi A, Winichagoon P, Dhanamitta S. Community-based surveillance for action towards health and nutrition: experience in Thailand. *Food and Nutrition Bulletin* 1995; **16**.	Scientific paper	Community-based nutrition surveillance

Doc Id	Reference	Type of document	Term used for CBS
119	Rehn M, Carnahan A, Merk H, Kühlmann-Berenzon S, Galanis I, Linde A, et al. Evaluation of an Internet-based monitoring system for influenza-like illness in Sweden. *PLoS ONE* 2014; **9**: e96740.	Scientific paper	No term
120	Ghebreyesus TA, Witten KH, Getachew A, Yohannes AM, Tesfay W, Minass M, et al. The community-based malaria control programme in Tigray, northern Ethiopia. A review of programme set-up, activities, outcomes and impact. *Parassitologia* 2000; **42**: 255–90.	Report	Community-based surveillance
121	Admon AJ, Bazile J, Makungwa H, Chingoli MA, Hirschhorn LR, Peckarsky M, et al. Assessing and improving data quality from community health workers: a successful intervention in Neno, Malawi. *Public Health Action* 2013; **3**: 56–9.	Scientific paper	No term
122	Merk H, Kühlmann-Berenzon S, Bexelius C, Sandin S, Litton J-E, Linde A, et al. The validity of self-initiated, event-driven infectious disease reporting in general population cohorts. *PLoS ONE* 2013; 8: e61644.	Scientific paper	Population based surveillance
123	Lamunu M, Lutwama JJ, Kamugisha J, Opio A, Nambooze J, Ndayimirije N, et al. Containing a haemorrhagic fever epidemic: the Ebola experience in Uganda (October 2000-January 2001). *Int J Infect Dis* 2004; **8**: 27–37.	Scientific paper	Community-based surveillance
124	Ngabo F, Nguimfack J, Nwaigwe F, Mugeni C, Muhoza D, Wilson DR, et al. Designing and Implementing an Innovative SMS-based alert system (RapidSMS-MCH) to monitor pregnancy and reduce maternal and child deaths in Rwanda. *Pan Afr Med J* 2012; **13**:31.	Scientific paper	No term
125	Community-based surveillance: guiding principles. Geneva: International Federation of Red Cross and Red Crescent Societies, 2017.	Guidance document	Community-based surveillance<br>Community event-based surveillance<br>Community-based health surveillance
126	Wanderley DM. Entomological surveillance of Chagas' disease in the State of Sao Paulo. *Rev Saude Publica* 1991; **25**: 28–32.	Scientific paper	No term
127	Qomariyah SN. A community-based surveillance system for maternal deaths in Indonesia. University of Aberdeen, 2013.	Thesis	Community-based surveillance
128	Brieger WR, Kendall C. Learning from local knowledge to improve disease surveillance: perceptions of the guinea worm illness experience. *Health Educ Res* 1992; **7**: 471–85.	Scientific paper	Market-based case detection
129	Ghebreyesus A, Alemayehu T, Bosma A, Hanna Witten K, Teklehaimanot A. Community participation in malaria control in Tigray region Ethiopia. *Acta Tropica* 1996; 61: 145–56.	Scientific paper	No term
130	Issah K, Nartey K, Amoah R, Bachan EG, Aleeba J, Yeetey E, et al. Assessment of the usefulness of integrated disease surveillance and response on suspected ebola cases in the Brong Ahafo Region, Ghana. *Infect Dis Poverty* 2015; **4**: 17.	Scientific paper	Community-based surveillance
131	Bhatia S, Dranyi T, Rowley D. A social and demographic study of Tibetan refugees in India. *Soc Sci Med* 2002; **54**: 411–22.	Scientific paper	Community-based surveillance
132	Sam-Abbenyi A, Dama M, Graham S, Obate Z. Dracunculiasis in Cameroon at the threshold of elimination. *Int J Epidemiol* 1999; **28**: 163–8.	Scientific paper	No term
133	Jones AH, Becknell S, Withers PC, Ruiz-Tiben E, Hopkins DR, Stobbelaar D, et al. Logistics of Guinea worm disease eradication in South Sudan. *Am J Trop Med Hyg* 2014; **90**: 393–401.	Scientific paper	Community-based surveillance
134	Technical Guidelines for Integrated Disease Surveillance and Response in the African Region. Brazzaville: World Health Organization Regional Office for Africa and Centers for Disease Control and Prevention, 2010.	Guidance document	Community-based surveillance

List and description of unique CBS systems identified

CBS Id	Doc Id	Country	Coverage	Setting	Start Year	Status CBS	Ongoing or end year	Purpose of CBS System	Scope	Data collection actor	Actor Selection Process	Actor Training duration	Actor Payment / Incentives	Data Collection Method	Data Reporting Method	Data Reporting Frequency	Report Recipient
1	110	Australia	Whole country	Rural; Urban	2006	Ongoing	2011	Monitoring	Acute respiratory illness	General community members				Self collection and reporting	Online via website	Weekly	
2	50, 53	Bangladesh	Limited area	Rural	2003 - 2004	End	2005 - 2006	Monitoring; Early detection	Unspecified newborn illness	Locally recruited cadres		35 days		House to house visit	Visit to or from supervisor	Fortnight	Non-government authority<br>Local level
3	89	Bangladesh	Limited area	Rural	1966	Ongoing	1989		Unspecified disease or syndrome; Birth; Death	Locally recruited cadres				House to house visit			
4	55	Benin	Limited area	Rural	2006	End	2010	Monitoring	Birth; Maternal death; Infant death	Locally recruited cadres	Local health care staffs select						Government health authority<br>Local level
5	115	Brazil	Limited area	Urban				Early detection	Triatomine bugs	General community members				Self collection and reporting		Ad hoc	Government health authority<br>Local level
6	90	Brazil	Limited area	Rural, Urban	1981	Ongoing	1993	Early detection	Triatomine bugs	General community members				Self collection and reporting	Visit to or from supervisor	Ad hoc	Non-government authority<br>Local level
7	95	Brazil	Limited area	Urban	2010	End	2014	Monitoring Early detection	Triatomine bugs	General community members				Self collection and reporting	Visit to or from supervisor	Ad hoc	Government health authority Local level
8	126	Brazil	Limited area		1984	Ongoing	1986		Triatomine bugs	General community members				Self collection and reporting	Visit to or from supervisor	Ad hoc	
9	87	Brazil	Whole country		1998	Ongoing	2005		Birth; Death	Locally recruited cadres				House to house visit		Monthly	
10	51	Cambodia	Limited area	Rural	2010	End	2011	Monitoring	Malaria	Locally recruited cadres				Visit by community people	Visit to or from supervisor		Government health authority<br>Local level

CBS Id	Doc Id	Country	Coverage	Setting	Start Year	Status CBS	Ongoing or end year	Purpose of CBS System	Scope	Data collection actor	Actor Selection Process	Actor Training duration	Actor Payment / Incentives	Data Collection Method	Data Reporting Method	Data Reporting Frequency	Report Recipient
11	40, 60	Cambodia	Limited area	Rural	2000	Ongoing	2005	Monitoring; Early detection	Malaria; Diarrhoeal disease; Measles; Viral haemorrhagic fever; Chronic cough; Birth; Death	Locally recruited cadres	General community people select,<br>Local health care staffs select	3 days		House to house visit	Visit to or from supervisor	Ad hoc, Monthly	Government health authority<br>Local level
12	132	Cameroon	Limited area		1990	End	1995	Monitoring	Guinea-worm disease	Locally recruited cadres	General community people select	6 days	Paid		Visit to or from supervisor	Weekly, Fortnight, Monthly, Every three months	Government health authority<br>Local level
13	39	Chad	Limited area	Refugee settlement	2004			Monitoring Early detection	Birth; Death	Locally recruited cadres				House to house visit,<br>Notification from community people	Visit to or from supervisor	Weekly	Non-government authority<br>Local level
14	59	Democratic Republic of the Congo	Limited area		2011	End	2011		Measles	Locally recruited cadres				House to house visit			
15	3	Democratic Republic of the Congo			2003	End	2005		Nutritional status of children	Locally recruited cadres	General community people select		Unpaid Programme-based allowances	Visit by community people		Monthly	Government health authority<br>Intermediary level
16	47	Ecuador	Limited area	Rural				Early detection	Yaws	Locally recruited cadres						Ad hoc	Government health authority<br>Local level
17	91	El Salvador			2008	Ongoing	2012	Early detection	Triatomine bugs	General community members				Self collection and reporting	Visit to or from supervisor	Ad hoc	Government health authority<br>Local level

CBS Id	Doc Id	Country	Coverage	Setting	Start Year	Status CBS	Ongoing or end year	Purpose of CBS System	Scope	Data collection actor	Actor Selection Process	Actor Training duration	Actor Payment / Incentives	Data Collection Method	Data Reporting Method	Data Reporting Frequency	Report Recipient
18	29, 48, 111, 112, 113, 119	Netherlands, Belgium, Portugal, Italy, United Kingdom, Sweden, France, Spain, Ireland, Denmark	Whole country	Rural; Urban	2010	End	2013	Monitoring	Acute respiratory illness	General community members				Self collection and reporting	Online via website	Weekly	Non-government authority<br>Central level
19	2, 9, 43, 109	Ethiopia	Limited area		2003	Ongoing	2014	Monitoring	Acute flaccid paralysis; Measles; Neonatal tetanus	Locally recruited cadres	Prominent community people suggest,<br>Local health care staffs select	3 days	Unpaid Gowns,<br>Umbrellas embossed with the project logo,<br>Bags,<br>Educational materials	House to house visit,<br>Actively contact community people	Visit to or from supervisor	Ad hoc, Monthly	Government health authority<br>Local level
20	67, 97	Ethiopia	Limited area	Rural	2012	End	2013	Monitoring	Birth; Death	Locally recruited cadres		12 months				Monthly	Non-government authority<br>Local level
21	93	Ethiopia	Limited area	Rural	1989	End	1990		Birth; Death	Locally recruited cadres			Paid			Monthly	Government health authority<br>Local level
22	120, 129	Ethiopia	Limited area	Rural	1992	Ongoing	1999	Monitoring	Malaria	Locally recruited cadres	General community people select	7 days	Unpaid		Visit to or from supervisor	Weekly, Monthly	Government health authority<br>Intermediary level
23	116	Ethiopia	Limited area	Rural	2010	End	2011	Monitoring	Birth; Death	Locally recruited cadres,<br>Specific group of community members		5 days		Notification from community people		Weekly	Government health authority<br>Local level

CBS Id	Doc Id	Country	Coverage	Setting	Start Year	Status CBS	Ongoing or end year	Purpose of CBS System	Scope	Data collection actor	Actor Selection Process	Actor Training duration	Actor Payment / Incentives	Data Collection Method	Data Reporting Method	Data Reporting Frequency	Report Recipient
24	5, 14, 25, 42, 46, 66, 78, `130	Ghana	Whole country	Rural; Urban	1997	Ongoing	2015	Monitoring; Early detection	Acute flaccid paralysis; Meningitis; Measles; Neonatal tetanus; Guinea-worm disease; Buruli Ulcer; Birth; Death; Maternal death; Infant death; Unusual health events	Locally recruited cadres	Prominent community people select		Unpaid<br>Programme-based allowances	House to house visit,<br>Notification from community people	Phone call,<br>Visit to or from supervisor	Ad hoc, Weekly, Fortnight, Monthly,	Government health authority<br>Local level
25	23, 91	Guatemala	Limited area	Rural	2003	Ongoing	2012	Early detection	Triatomine bugs	General community members				Self collection and reporting	Visit to or from supervisor	Ad hoc	Government health authority<br>Local level
26	32, 63, 64	Guatemala	Limited area	Rural; Urban	1958	Ongoing	1992	Monitoring; Early detection	Malaria	Locally recruited cadres	Prominent community people suggest,<br>General community people select,<br>General community people select through voting	2 days	Unpaid Christmas cards,<br>Calendar,<br>Preferential treatment in health care services,<br>Exemption from yearly municipal tax	Visit by community people	Visit to or from supervisor	Weekly	Government health authority<br>Local level
27	125	Haiti			2014			Monitoring; Early detection	Diarrhoeal disease	Locally recruited cadres					SMS	Daily	
28	49, 91	Honduras			2008	Ongoing	2012	Early detection	Triatomine bugs	General community members				Self collection and reporting	Visit to or from supervisor	Ad hoc	Government health authority<br>Local level
29	107	Honduras	Limited area		1999	Ongoing	2003	Monitoring	Pregnancy complications; Neonatal death	Specific group of community members		3 days				Monthly	Non-government authority<br>Local level

CBS Id	Doc Id	Country	Coverage	Setting	Start Year	Status CBS	Ongoing or end year	Purpose of CBS System	Scope	Data collection actor	Actor Selection Process	Actor Training duration	Actor Payment / Incentives	Data Collection Method	Data Reporting Method	Data Reporting Frequency	Report Recipient
30	13	India	Limited area			Ongoing	2007	Monitoring	Birth; Death	Locally recruited cadres				House to house visit, Actively contact community people			
31	4	India	Limited area	Rural	2005	End	2005	Monitoring; Early detection	Diarrhoeal disease; Malaria; Measles; Viral haemorrhagic fever; Meningitis; Acute respiratory illness; Tuberculosis; Acute flaccid paralysis; Birth; Death	Specific group of community members						Weekly	Government health authority Local level
32	1	India	Limited area	Rural	1980	Ongoing	1987	Monitoring Early detection	Neonatal death	Locally recruited cadres, Specific group of community members				House to house visit			Government health authority Local level
33	92	India	Limited area	Rural	1983	Ongoing	2004		Birth; Death	Locally recruited cadres						Ad hoc	Government health authority Local level
34	65	India	Limited area					Early detection	Unusual health events	Locally recruited cadres				Notification from community people	Phone call	Ad hoc	Government health authority Local level
35	131	India	Limited area	Refugee settlement; Monastries	1994	Ongoing	1996	Monitoring	Unspecified disease or syndrome; Birth; Death; Immunisation status of children	Locally recruited cadres, Specific group of community members				House to house visit		Every three months	Government health authority Central level

CBS Id	Doc Id	Country	Coverage	Setting	Start Year	Status CBS	Ongoing or end year	Purpose of CBS System	Scope	Data collection actor	Actor Selection Process	Actor Training duration	Actor Payment / Incentives	Data Collection Method	Data Reporting Method	Data Reporting Frequency	Report Recipient
36	26	Indonesia	Limited area	Rural	1972				Malaria; Smallpox; Diarrhoeal disease; Birth; Death;	Locally recruited cadres				House to house visit, Actively contact community people			
37	127	Indonesia	Limited area	Rural	2008	End	2008	Monitoring	Birth; Maternal death; Infant death	Locally recruited cadres			Unpaid		Visit to or from supervisor		Government health authority, Government non-health authority Local level
38	98	Indonesia	Whole country	Rural, Urban	1984	Ongoing	2016		Nutritional status of children	Locally recruited cadres				Visit by community people		Monthly	Government health authority Local level
39	101	Kenya		Rural					Birth; Death	Locally recruited cadres							
40	27, 61,	Laos	Whole country			Ongoing	2014		Malaria; Birth; Death	Locally recruited cadres	Prominent community people select, Local health care staffs select		Unpaid Free health service, Programme-based allowances		Visit to or from supervisor	Monthly	Government health authority Local level, Intermediary level
41	8, 28, 33, 81, 121	Malawi	Whole country	Rural	1995	Ongoing	2014	Monitoring; Early detection	Unspecified disease or syndrome; Birth; Death	Locally recruited cadres		12 weeks	Paid	House to house visit	Visit to or from supervisor	Monthly	
42	67, 75	Malawi	Limited area		2010	End	2013	Monitoring	Birth; Death	Locally recruited cadres			Paid			Monthly	
43	24	Malaysia	Limited area		1987			Early detection	Malaria	Locally recruited cadres	General community people select		Unpaid Merit certificate, Preferential treatment in health care services	Visit by community people	Visit to or from supervisor	Weekly, Fortnight, Monthly, Every three months	Government health authority Local level

CBS Id	Doc Id	Country	Coverage	Setting	Start Year	Status CBS	Ongoing or end year	Purpose of CBS System	Scope	Data collection actor	Actor Selection Process	Actor Training duration	Actor Payment / Incentives	Data Collection Method	Data Reporting Method	Data Reporting Frequency	Report Recipient
44	67, 82	Mali	Limited area	Rural	2012	End	2013	Monitoring	Birth; Death	Locally recruited cadres		3 days				Monthly	
45	30	Mozambique	Limited area	Refugee settlement					Birth; Death; Immunisation status of children	Locally recruited cadres			Unpaid Tshirt		Visit to or from supervisor	Fortnight	Non-government authority
46	99	Mozambique	Limited area	Rural	2000	End	2003		Unspecified disease or syndrome; Birth; Death	Locally recruited cadres				House to house visit		Monthly	Government health authority<br>Local level
47	70, 77	Nepal	Limited area	Rural		Ongoing	2015		Unspecified disease or syndrome	Locally recruited cadres			Unpaid<br>Travel allowances			Twice a week	Non-government authority<br>Local level
48	62	Nepal	Limited area	Rural					Birth	Locally recruited cadres	General community people select						
49	22, 128	Nigeria	Limited area	Rural; Urban	1990	End	1991	Early detection	Guinea-worm disease	Locally recruited cadres	General community people select				Visit to or from supervisor	Weekly	Non-government authority
50	105	Pakistan	Whole country		1994	Ongoing	2008	Monitoring	Birth; Death; Maternal death; Infant death; Immunisation status of children; Low birth weight; Use of family planning methods	Locally recruited cadres						Monthly	Government health authority<br>Local level
51	114	Paraguay			2002	Ongoing	2003		Triatomine bugs	Specific group of community members,<br>General community members				Self collection and reporting		Ad hoc	Government non-health authority<br>Local level

CBS Id	Doc Id	Country	Coverage	Setting	Start Year	Status CBS	Ongoing or end year	Purpose of CBS System	Scope	Data collection actor	Actor Selection Process	Actor Training duration	Actor Payment / Incentives	Data Collection Method	Data Reporting Method	Data Reporting Frequency	Report Recipient
52	108	Philippines	Limited area	Urban	1984	Ongoing	1985	Monitoring	Unspecified disease or syndrome;<br>Birth;<br>Death;<br>Use of family planning methods;<br>Nutritional status of children;<br>Immunisation status of children	Locally recruited cadres,<br>General community members				Self collection and reporting	Visit to or from supervisor	Weekly, Every three months	Government health authority<br>Local level
53	12	Rwanda	Whole country	Rural	2004	Ongoing	2006	Early detection	Nutritional status of children	Locally recruited cadres		3-5 days	Unpaid	Visit by community people			
54	124	Rwanda	Limited area		2010	Ongoing	2012	Early detection	Maternal death;<br>Infant death;<br>Pregnancy complications	Locally recruited cadres		2 days	Unpaid		SMS	Ad hoc	
55	71	Rwanda	Whole country	Rural		Ongoing	2012		Tuberculosis;<br>Death;<br>Nutritional status of children;<br>Use of family planning methods	Locally recruited cadres				House to house visit		Monthly	
56	58	Senegal	Limited area		2006				Birth;<br>Death;<br>Maternal death;<br>Neonatal death	Locally recruited cadres				House to house visit	SMS, Mobile phone application	Ad hoc	Non-government authority
57	17, 20, 21, 41, 74, 83	Sierra Leone	Limited area	Rural; Urban	2015	Ongoing	2015	Early detection	Viral haemorrhagic fever	Locally recruited cadres	Prominent community people suggest,<br>Local health care staffs select		Unpaid<br>Travel allowances	House to house visit,<br>Actively contact community people	Phone call	Ad hoc	Non-government authority<br>Local level

CBS Id	Doc Id	Country	Coverage	Setting	Start Year	Status CBS	Ongoing or end year	Purpose of CBS System	Scope	Data collection actor	Actor Selection Process	Actor Training duration	Actor Payment / Incentives	Data Collection Method	Data Reporting Method	Data Reporting Frequency	Report Recipient
58	125	Sierra Leone	Limited area		2015	Ongoing	2017	Monitoring; Early detection	Viral haemorrhagic fever; Diarrhoeal disease; Measles; Death	Locally recruited cadres					SMS	Ad hoc	Non-government authority<br>Local level
59	19, 133	South Sudan	Limited area	Rural	2006	Ongoing	2012	Monitoring; Early detection	Guinea-worm disease	Locally recruited cadres				House to house visit	Visit to or from supervisor	Daily, Weekly	Government health authority<br>Local level
60	43	South Sudan	Limited area	Rural	2010	Ongoing	2015	Monitoring	Acute flaccid paralysis; Measles; Neonatal tetanus	Locally recruited cadres			Unpaid<br>Gowns,<br>Umbrellas embossed with the project logo,<br>Bags,<br>Educational materials	House to house visit,<br>Actively contact community people		Monthly	Government health authority<br>Local level
61	73	Sweden	Limited area	Rural; Urban	2013	End	2014	Monitoring	Diarrhoeal disease; Acute respiratory illness	General community members				Self collection and reporting	Online via website	Weekly	Government health authority<br>Central level
62	122	Sweden	Limited area	Urban	2007	Ongoing	2009	Monitoring	Acute respiratory illness	General community members				Self collection and reporting	Online via website, Phone call	Ad hoc, Weekly	
63	37, 80	Tanzania	Limited area	Urban	2004	Ongoing	2012	Monitoring Early detection	Mosquito and its habitat	Locally recruited cadres	Prominent community people select		Paid	House to house visit		Daily, Weekly	Government non-health authority<br>Local level
64	7	Tanzania	Limited area	Rural				Monitoring	Maternal death; Neonatal death; Infant death	Locally recruited cadres							
65	76	Tanzania	Limited area	Rural	1983	End	1989	Monitoring	Nutritional status of children; Under-five death	Locally recruited cadres							Government non-health authority<br>Local level

CBS Id	Doc Id	Country	Coverage	Setting	Start Year	Status CBS	Ongoing or end year	Purpose of CBS System	Scope	Data collection actor	Actor Selection Process	Actor Training duration	Actor Payment / Incentives	Data Collection Method	Data Reporting Method	Data Reporting Frequency	Report Recipient
66	31	Tanzania	Limited area	Rural					Birth; Death	Locally recruited cadres	General community people select, General community people select through voting	9 months					
67	10	Thailand	Whole country	Rural	1961	Ongoing	1989	Monitoring; Early detection	Malaria	Locally recruited cadres				Visit by community people			
68	86	Thailand	Whole country	Rural	2004	Ongoing	2007	Early detection	Acute respiratory illness	Locally recruited cadres	Prominent community people suggest, Local health care staffs select	23 days	Unpaid Free health service	House to house visit, Notification from community people	Phone call, Wireless radio	Ad hoc, Daily, Fortnight	Government health authority Local level
69	104, 118	Thailand	Whole country	Rural	1982	Ongoing	1995	Monitoring	Nutritional status of children	Locally recruited cadres, Specific group of community members				Visit by community people		Monthly, Every three months, Twice a year	Government health authority, Government non-health authority Local level, Intermediary level
70	57	Thailand	Limited area	Rural					Diarrhoeal disease	Locally recruited cadres				House to house visit, Notification from community people			
71	88	Tunisia	Limited area	Rural	2009	Ongoing	2014		Cutaneous Leishmaniasis	Specific group of community members							Government health authority Local level
72	34	Uganda	Limited area	Rural				Monitoring	Unspecified newborn illness	Locally recruited cadres	General community people select	5 days	Unpaid	House to house visit			Government health authority Local level

CBS Id	Doc Id	Country	Coverage	Setting	Start Year	Status CBS	Ongoing or end year	Purpose of CBS System	Scope	Data collection actor	Actor Selection Process	Actor Training duration	Actor Payment / Incentives	Data Collection Method	Data Reporting Method	Data Reporting Frequency	Report Recipient
73	123	Uganda	Limited area	Rural	2000	Ongoing	2002	Early detection	Measles; Meningitis; Viral haemorrhagic fever	Locally recruited cadres, Specific group of community members						Ad hoc	Government health authority Intermediary level
74	52	United States	Limited area	Rural	2006	Ongoing	2014	Early detection	Suicidal and self-injurious behavior	General community members				Notification from community people	Phone call, Fax	Ad hoc	Non-government authority Central level
75	56	Vanuatu	Limited area	Rural	1992	Ongoing	2010	Early detection	Malaria	Locally recruited cadres							
76	44	Vietnam		Rural	2007			Early detection	Acute respiratory illness	Locally recruited cadres				House to house visit	Visit to or from supervisor	Monthly	Government health authority Local level
77	68	Zambia	Limited area	Rural; Urban		Ongoing	2014	Monitoring	Malaria	Locally recruited cadres		2-4 days		House to house visit	Mobile phone application	Ad hoc	Government health authority Intermediary level
78	100	Zambia	Limited area	Rural	1982			Monitoring	Unspecified disease or syndrome; Birth; Death; Nutritional status of children	Locally recruited cadres, Specific group of community members, General community members				House to house visit, Notification from community people, Self collection and reporting		Monthly, Twice a year	Government health authority, Non-government authority Local level
79	117	Zimbabwe	Whole country	Rural		Ongoing	1981		Unspecified disease or syndrome; Birth; Death	Locally recruited cadres	General community people select	8 weeks				Monthly	Government health authority Local level
