# Supplementary material for: Community-based surveillance: A scoping review
Source: PLoS One. 2019 Apr 12;14(4):e0215278. doi: 10.1371/journal.pone.0215278 (PMC6461245; doi:10.1371/journal.pone.0215278)
Supplement: S1 Text — (PDF) [file pone.0215278.s003.pdf]

Supporting text 1 – Summary of available guidance and recommendations for community-based surveillance.

A summary of guidance and recommendations provided on different topics of interest for community-based surveillance (CBS) are presented in the following sections.

Setting

The following criteria were proposed to identify where to implement a CBS system:

- Setting where the use of community structures increases the likelihood that events will be reported [1,2].
- Setting where the following minimum requirements are available before implementing a CBS system [1]: possibility of community engagement; feasibility (time, financial resources, manpower, motivation); possibility that reported events lead to a response.

Purpose

Community-based surveillance was expected to:

- increase awareness of the symptoms of a disease or condition and encourage its self-reporting by the community [2];
- notify health alerts [1];
- early detect and respond to public health events [3,4];
- lead to a response [5];
- monitor the health status of a population [3,4].

Scope

Proposed diseases and events to be put under surveillance were:

- Epidemic prone diseases such as cholera, influenza, meningococcal meningitis and viral haemorrhagic fever; diseases targeted for elimination and eradication such as polio and measles; diseases of public health importance and unusual events [3,4].
- Disease for which extremely high sensitivity is essential [2].

In the 2017 IFRC guide [1], two strategies are proposed: the reporting of unusual events; or the reporting of the numbers of one or several specific disease(s) using community case definitions.

In any case, the scope of the CBS system should address the local needs [1,3,4]. Reviewing the disease trends for previous years and the causes of deaths in the community was proposed as a first step to define the scope of the CBS system [1].

Process for CBS implementation

Several activities have been described for the implementation of a CBS system:

- Planning phase:
  - Involve health authorities [6].
  - Select communities according to specific criteria [6].
  - Identify information needs for decision-making, including community problems and available resources [1,7].
  - Get information on existing surveillance networks [1].
  - Select a focal point in charge of the CBS system [3,4,6].
  - Identify community resource persons and/or community volunteers for CBS [3,7,8].
  - Establish objectives of the CBS system [1,9].
  - Define the events to be reported and the frequency of reporting [1,3].
  - Design the CBS system [7].
  - Develop data collection instruments [9].
  - Develop a data analysis plan on how to analyse and disseminate the information [1].
  - Develop a CBS plan of action [3].
- Implementation phase:
  - Ensure expertise and resources for the functioning of the CBS system [1,6,7].
  - Field test CBS instruments [9].
  - Train CBS focal points and community resource persons [3,6,8,10].

- Engage community resource persons and/or community volunteers for CBS [8,10].
- Explain and make known the CBS system in the community [8].
- Disseminate simplified case definitions for community use [3,8].
- Assure use of analysis and interpretation [9].
- Assure use of regular feedback to the community [3,6,8].
- Include routine monitoring indicators in the supervisory instruments [6].
- Monitor and evaluate the implementation of the CBS activities [3].

In some documents, the community was required to be involved at all steps of CBS implementation and operation [7,11], in other documents health facilities were expected to play an important role for CBS in their health area [3,4].

Activities of CBS

The main activities to be performed under CBS were described as:

- Identify cases and events [1,3,10].
- Report cases and events to the next level [1,3,10].
- Integrate information from CBS into the regular public health surveillance system [10].
- Analyse and interpret findings [3].
- Investigate and confirm cases and events [1,3].
- Communicate and provide feedback to the community [1,3,4,10].
- Conduct preparedness and response activities [3,10].
- Participate in verbal autopsies to determine causes of death [3].
- Organize collective preventive activities [10].
- Evaluate and improve the CBS system [3].

Responsibilities of different actors for some of the CBS activities were proposed:

- Surveillance actors:
  - Identify cases, events or outbreaks and report it to the next level in a timely manner [3,4,10].
  - Participate and support in outbreak investigation, contact tracing, risk mapping [3,4], and prevention and response activities [3,4,10].
- CBS supervisors: support community informants, community members, and local health facilities in different activities relating to CBS [3,4,10] such as:
  - Bridge the information flow between community and health authorities [10].
  - Filter/screen alerts obtained from community informants before sending them to the next level [1,12].
- Health authorities at local and intermediate level:
  - Respond to reports by community informants [10,12].
  - Integrate information from CBS into the regular public health surveillance system [10].

Actors in charge of data collection

Two different modalities of community involvement were proposed for data collection [13]: collection and reporting by general community members, or collection and reporting by selected community members.

In the latter case, the selection of the surveillance actors directly by the community was recommended in several documents [7,10,11]. Before selecting data collection actors, communities were to be well informed about the CBS system, the expected job of the data collection actors, and their ideal profile.[10] The proposed desirable qualities for a community data collection actor were:

- respected and accepted by the community [3,10];
- honest, mature, stable [14];
- respectful of people's beliefs and traditional practices [10];
- interested in health and community work [10];
- motivated to work for the community's welfare [14];
- willing to learn new things [10];
- being a good communicator [10];
- likely to be aware of events occurring in the community [1];
- supported by his or her family [10].

The following factors were suggested to be taken into account to determine the optimal number of selected actors in a community [1]:

- the method used for data collection (active or passive data collection),

- the distribution of households in the community (scattered or clustered),
- the ease in assessing the community (including available means of transportation and communication).

Given examples of optimal number of selected actors ranged from 3 to 5 per community [10] or one per 500 community members [1]. Specific types of community informants were proposed to be especially considered, such as pharmacists, school teachers, local leaders, religious leaders, traditional healers, traditional birth attendants or already existing community health workers [3,4,13,15].

Once selected, data collection actors should be trained [1,3,4,10]. Several methodologies could be used for the training, e.g. lectures, demonstrations, role play, discussions [4,10]. The appropriate number of participants in a training should be defined taking into account the total number of actors and how close they live from each other [4,10], e.g. a maximum of 30 participants per session [4]. Trainings were to be conducted in locations and schedules convenient for participants [10]. Proposed topics were:

- identification of diseases, conditions and public health events to be collected at the community level [3,4];
- structure of community-based surveillance (reporting structures, selection modality, roles and responsibilities of actors) [4];
- recording and reporting modalities for identified cases/events [3,4];
- investigation of events [4];
- supervision, monitoring and evaluation of the CBS system [4];
- provision of feedback to the community [4].

The level of training should depend on the previous knowledge of the actors and the type of CBS being implemented [1].

Data collection

The modalities of data collection and reporting were recommended to be simple, purposeful and easy to set up [1,9,16]. For Rosales et. al. information collected by CBS needs to be related to action to avoid overburdening the system [9].[9] To ensure only necessary information is collected, the 2017 IFRC guide [1] proposes that no more than four to eight community case definitions should be used in the CBS system. These community case definitions should be easy to understand, read and report, and they or their order should remain unchanged unless to adjust to a major change in the CBS system [1]. Simple community case definitions were available for several diseases, syndromes and events:

- Acute flaccid paralysis [3,4,10,15].
- Acute watery diarrhea [3,4,15].
- Acute jaundice [1,3,4,10,15].
- Acute respiratory infection [1].
- Adverse event following immunization [3,4,15].
- Cholera [1,3,4,10,15].
- Diarrhoea with blood [1,3,4,15].
- Dracunculiasis [3,4,15].
- Hepatitis [1,3,4,15].
- Influenza-like illness [3,4,15].
- Leprosy [3,4,15].
- Malaria [3,4,15].
- Maternal death [3,4,15].
- Measles [1,3,4,10,15].
- Meningococcal meningitis [3,4,10,15].
- Neonatal death [3,4,15].
- Neonatal tetanus [10].
- Trachoma [3,4,15].
- Unusual event [1].

Data reporting

Two frequencies of data reporting were proposed [1,4]: immediate reporting as soon as a case or event is detected; or regular reporting on a routine basis with zero reporting. The 2010 technical guidelines for Integrated Disease Surveillance and Response in the African Region [15] recommend to report to the local community health-care institutions or the appropriate health personnel. Where available, using mobile technology for reporting was mentioned as a possible mean to lower the costs and increase timely reporting and response [1].

Several documents provide templates of reporting forms for: immediate reporting [4,10], daily reporting [4], weekly reporting [4], monthly reporting [17].

Feedback

While several documents mention the need to provide feedback to the community [1,3,4,10], few provide specific recommendations to do so.

In one guide [4], community-based surveillance focal points are required to seek guidance from the national level authorities on giving feedback. They are required not to address the community unless they are delegated to do so and to follow the directions and key messages provided by the national authorities. Among feedback modalities that may be recommended by authorities were: regular community briefings, regular meetings with local stakeholders, door-to-door campaigns.

One guide [10] provides specific practical recommendations in helping surveillance volunteers to provide feedback to communities mainly through community meetings.

Monitoring and evaluation

Several aspects to be monitored are proposed in the 2015 WHO CBS training manual [4]:

- Community-based surveillance and the agreed response activities are relevant, on track and being achieved.
- The role of community-based surveillance and the activities are well described.
- A community-based surveillance supervision plan exists.
- A checklist exists and is used to monitor how well the community-based surveillance focal points are carrying out the agreed surveillance functions.
- Reporting and record keeping functions of the CBS are timely and complete.
- The community-based surveillance detection and notification procedures for suspected public health events are functional.

In the same guide [4], supervisory visits are proposed to:

- ensure that:
  - appropriate community-based surveillance supplies such as forms and tally sheets are available and are used properly,
  - the required standard case definitions and guidelines are available,
  - the community-based surveillance focal points know how to use the community case definitions to report suspected public health events in their catchment area,
  - supervisory plans for improvement of surveillance and response are updated,
  - successful activities are recorded and encouragement for their continuation provided; and
- provide:
  - feedback to community-based surveillance focal points,
  - on-the-job training as needed if a problem is identified,
  - follow-up on requests for assistance,
  - feasible solutions for identified problems.

The 2017 IFRC guide proposes indicators to evaluate a CBS system:

- Number of cases, events and outbreaks detected.
- Number of events responded within 24/48 hours.
- Number of actors trained for surveillance.
- Coverage of the CBS system.
- Reporting rate.
- Impact the system had in the decline of cases

The same guide proposes the following approaches to collect feedback on the CBS system from the community [1]:

- Open and regular community meetings where all issues are noted and acted upon.
- Focus group discussions with volunteers and/or community leaders.
- Suggestions and complaints box(es) for use in the community.
- Appointment of a community representative(s) to gather feedback and complaints.
- A communication pipeline for feedback (e.g. SMS or hotline).

References

1. Community-based surveillance: guiding principles. Geneva: International Federation of Red Cross and Red Crescent Societies, 2017.

2. Curry D, Bisrat F, Coates E, Altman P. Reaching beyond the health post: Community-based surveillance for polio eradication. Dev Pract. 2013;23: 69–78.

3. Integrated disease surveillance and response in the African Region: a guide for establishing community based surveillance. Brazzaville: World Health Organization Regional Office for Africa, 2014.

4. Integrated Diseases Surveillance and Response in the African Region. Community-based Surveillance (CBS) Training Manual. Brazzaville: World Health Organization Regional Office for Africa, 2015.

5. Hashimoto K, Yoshioka K. Review: surveillance of Chagas disease. Adv Parasitol. 2012;79: 375–428.

6. BASICS II country report: Zambia. Arlington: Basic Support for Institutionalizing Child Survival Project (BASICS II) for the United States Agency for International Development, 2004.

7. Immink M. Community-based food and nutrition surveillance as an instrument of socio-economic development in Central America: a point of view. Food Nutr Bull. 1988;10: 13–5.

8. Ebola and Marburg virus disease epidemics: preparedness, alert, control, and evaluation. Geneva: World Health Organization, 2014.

9. Rosales A, Galindo J, Flores A. A community based surveillance system for maternal and early neonatal complications: the Intibuca case study. Baltimore: Catholic Relief Services, 2004.

10. Handbook for community surveillance coordinators to support community participation in detection and prevention of polio and other diseases. Version 2. Washington, D.C: Academy for Educational Development, CHANGE Project, 2001.

11. Ruebush TK, Godoy HA. Community participation in malaria surveillance and treatment. I. The Volunteer Collaborator Network of Guatemala. Am J Trop Med Hyg. 1992;46: 248–60.

12. Standard Operating Procedure for community event-based surveillance for Ebola virus disease in Sierra Leone. New York: International Rescue Committee, 2015.

13. A Guide to Establishing Event-based Surveillance. Manila: World Health Organization Western Pacific Region, 2008

14. Ruebush TK, Weller SC, Klein RE. Qualities of an ideal volunteer community malaria worker: a comparison of the opinions of community residents and national malaria service staff. Soc Sci Med. 1994;39: 123–31.

15. Technical Guidelines for Integrated Disease Surveillance and Response in the African Region. Brazzaville: World Health Organization Regional Office for Africa and Centers for Disease Control and Prevention, 2010.

16. Shayo E, Mboera LEG, MMbuji P, Rumisha SF, Senkoro KP, Mwami AJ. The role of community and traditional healers in communicable disease surveillance and management in Babati and Dodoma Districts, Tanzania. Tanzania Health Research Bulletin. 2003;5: 48–55.

17. Oum S, Chandramohan D, Cairncross S. Community-based surveillance: a pilot study from rural Cambodia. Trop Med Int Health. 2005;10: 689–97.
