# Supplementary material for: Community-based surveillance: A scoping review
Source: PLoS One. 2019 Apr 12;14(4):e0215278. doi: 10.1371/journal.pone.0215278 (PMC6461245; doi:10.1371/journal.pone.0215278)
Supplement: S2 Text — (PDF) [file pone.0215278.s004.pdf]

Supporting text 2 – Supplemental study on the usage of the term “community-based surveillance” in the literature.

Selection criteria

Inclusion criteria:

— Explicit mention in the title or abstract of the term “community-based surveillance”.

OR

— Explicit mention in the title or abstract of a term including “community based” and “surveillance” and other elaborative words in between (e.g. “community based sentinel surveillance”; “community event based surveillance”).

Exclusion criteria:

— Scope of the document unrelated to human health.

— Document in other language than English, French, Spanish and Portuguese.

— Unavailability of full text.

— Abstract of oral or poster presentation at a conference.

No publication time limit was used for the selection of studies.

Selection process

The title and abstract of all unique documents retrieved from the scoping review search were screened by one reviewer for the term “community-based” and “surveillance” with or without other elaborative words in between. Further screening of the selected documents was done during the data collection process (i.e. a document could be later excluded based on the full text review).

Data collection and analysis

One reviewer performed data collection using Google Forms (https://www.google.com/forms/about/). Information was collected for the following variables:

— Type of document.

— Term used related with “community-based surveillance”.

— Description of the approach termed as “community-based surveillance” in the document (Yes/No), if yes: source of data; data collector: type of actor collecting the information; purpose of the data collection.

All the analyses were performed using the R statistical software [1].

Results

Out of all records screened for the scoping review, we looked at 232 documents using the term “community-based surveillance” in their title or abstract (Fig 1).

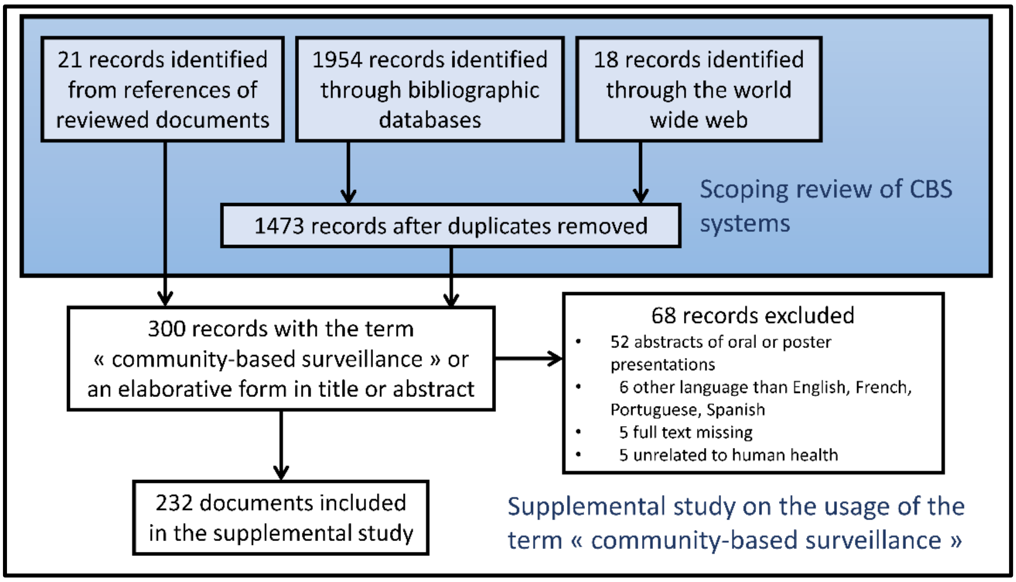

Fig 1. Identification of documents for the supplemental study on the usage of the term “community-based surveillance”

Out of the 232 documents, 178 (77%) described the approach they termed as "community-based surveillance" whereas 54 (23%) didn't provide any details.

The purpose of the approach termed as "community-based surveillance" was:

- research: n= 109/178, 61%;
- public health surveillance (monitoring of the health status of a population or early detection of public health risks): n= 69/178, 39%.

Information on the source of data used by the approach termed as "community-based surveillance" was provided in 177 documents:

- Community members: n=121/177 (68%),
  - from the whole community or relevant subgroups (e.g. families of children less than 5 years old): n=81/121 (67%);
  - from a sample, or enrolled members, of the community: n=40/121 (33%).
- Healthcare facilities (directly from patients or their records): n=41/177, 23%,
  - from all patients or relevant subgroups (e.g. children less than 5 years old): n=14/41 (34%);
  - from a sample of, or enrolled, patients: n=27/41 (66%).
- Community members and healthcare facilities: n= 10/177, 6%.
- Healthcare facilities and other sources (fire brigades, courthouses, work sites and child-care facilities): n=3/177, 2%.
- Community members and other sources (media and police): n = 2/177, 1%.

Out of 70 documents where data was collected from a sample of community members or patients, in 96% (n=67) the purpose of the approach termed as "community-based surveillance" was research (Fig 2).

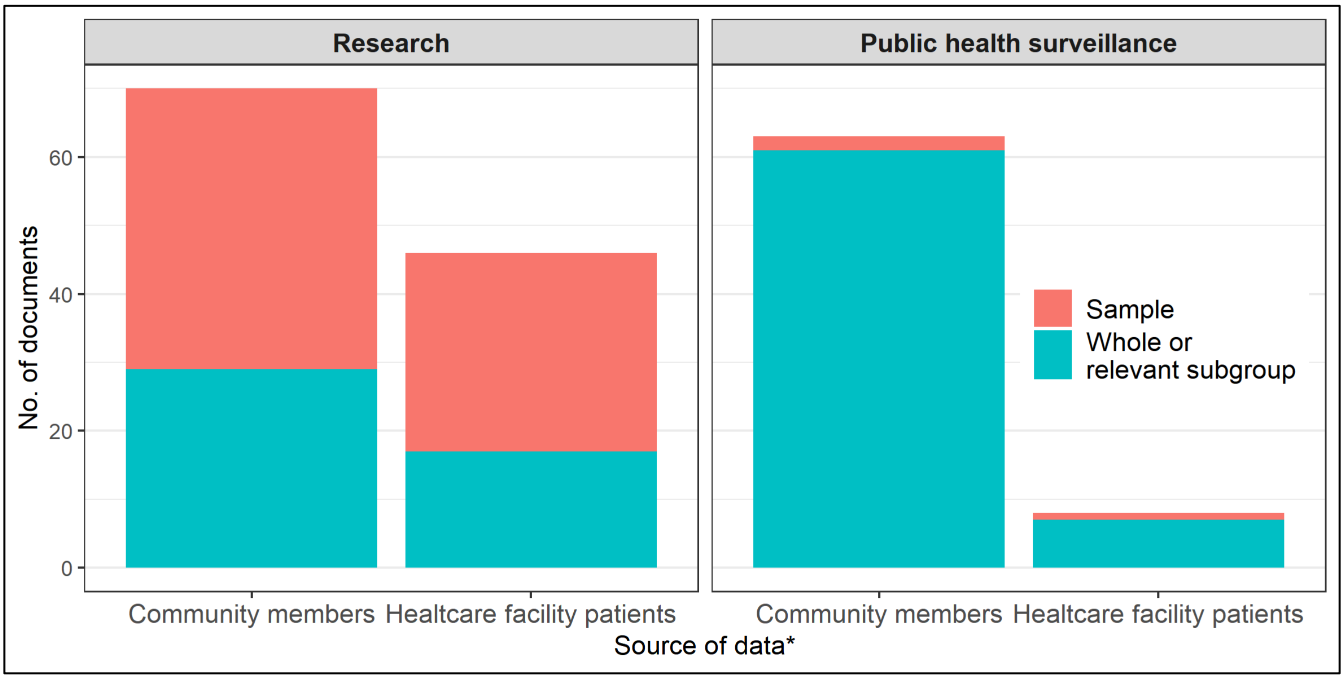

Fig 2. Distribution of the source of data used by the approach termed as "community-based surveillance" by its purpose

*Ten approaches used both community members and healthcare facility patients as source of data.

The actors in charge of collecting data for the approach termed as “community-based surveillance” are presented in Fig 3.

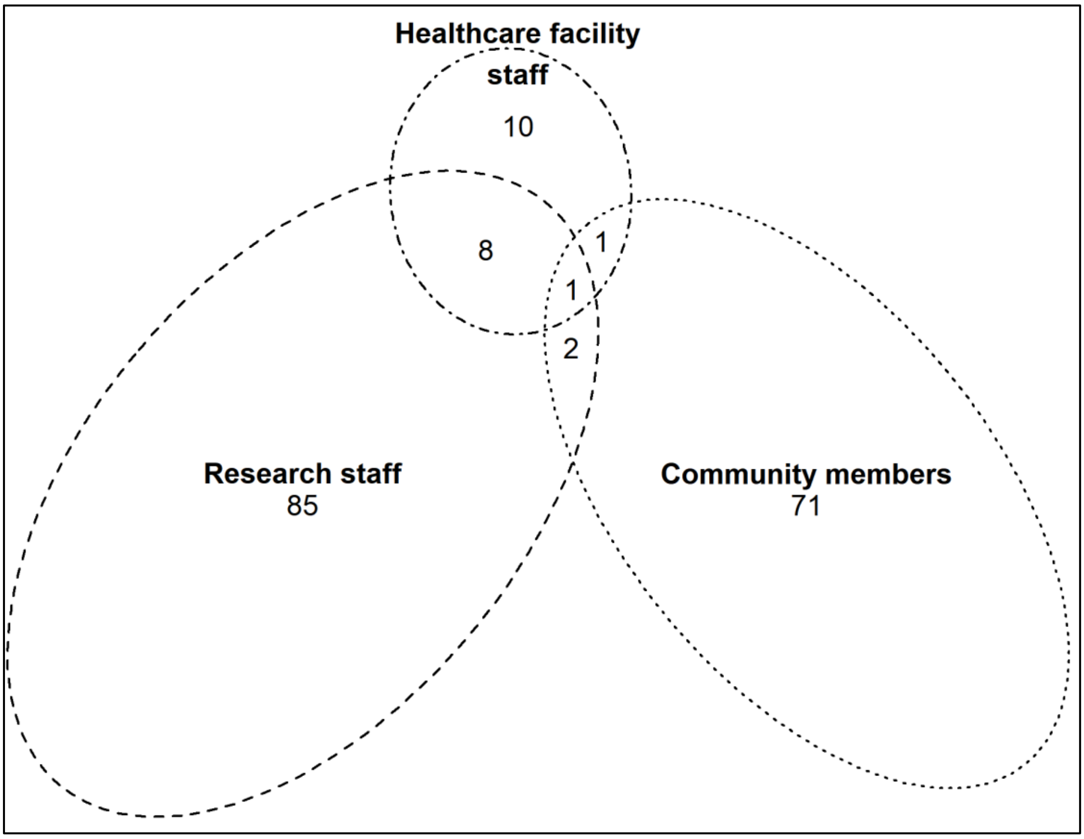

Fig 3. Actors in charge of data collection in the approaches termed as “community-based surveillance” (n=178)

Fig 4 presents the distribution of approaches termed as “community based surveillance” by their type of source of data, actors in charge of data collection and purpose.

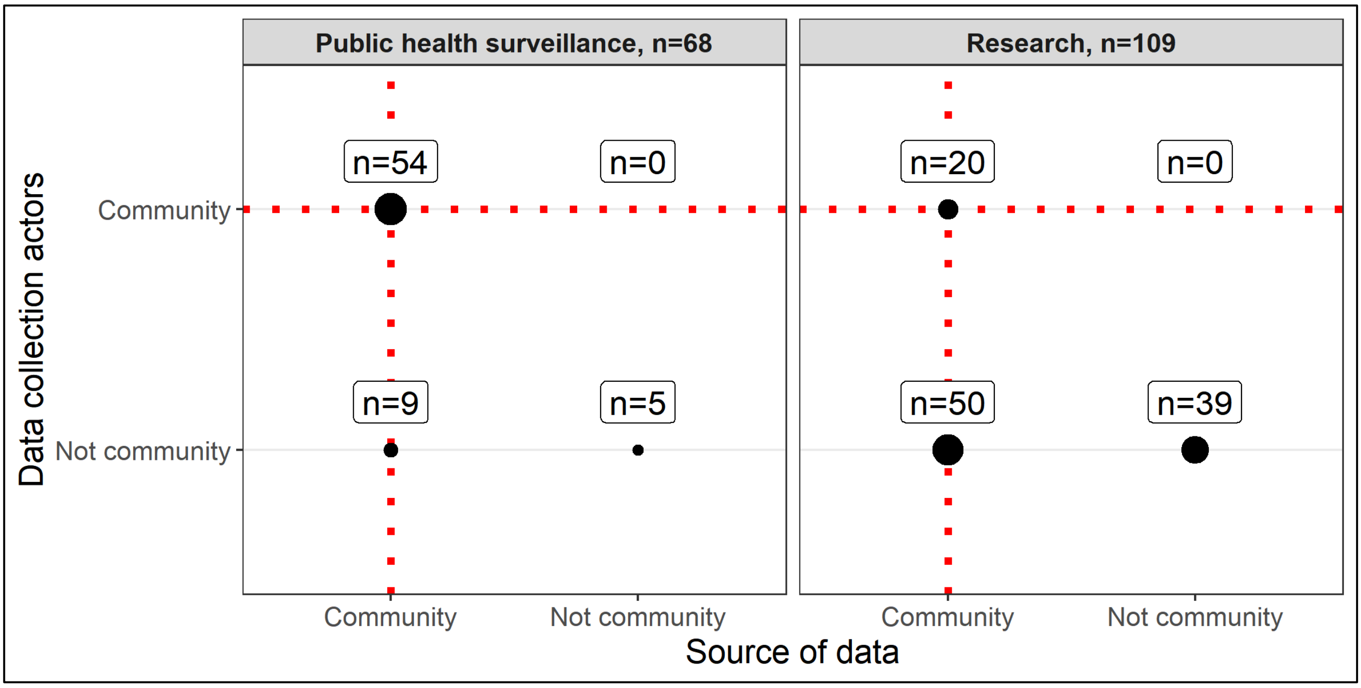

Fig 4. Distribution of approaches termed as “community based surveillance” by the type of source of data, actors in charge of data collection and purpose

Around one third of the documents used the term “community-based surveillance” to describe an approach where data was collected from the community by community members for public health surveillance purposes (31%, n=54/177). All except two, were included in our review of CBS systems. Out of two excluded documents, for one [2] there was collection but no reporting of information for public health surveillance, and for the other [3], CBS was discussed as one of the possible strategies for control of Buruli ulcer, without describing any specific CBS system.

- e.g. “*A CBSS [community based surveillance system] [..] developed and implemented since July 2000 in seven rural communes*“ where village health volunteers collected information on diseases/syndromes and vital events from community through home visits and reported it on an ad hoc and monthly manner to local health facilities to “*provide timely and representative information on major health problems and life events, and so permit rapid and effective control of outbreaks and communicable diseases in general*. [4]”

The second most frequent use of the term “community-based surveillance” was to denote a research design where information was collected from the community by surveyors or healthcare facility staff (28%, n=50/177).

- e.g. In a “longitudinal community-based surveillance of children from urban Mexico”, surveyors conducted home visits to collect child’s stool sample and interview mothers to “longitudinally assess the pattern of intestinal colonization by gram negative bacilli resistant to one or more of seven antimicrobial agents. [5]”

The third most frequent use of the term “community-based surveillance” met none of the criteria that we previously used to describe a CBS system (22%, n=39/177). They generally described a specific research study where surveyors collected data on a specific sample of enrolled patients.

- e.g. “enhanced, community-based surveillance in […] Salvador, Brazil to identify acute febrile illness patients with laboratory evidence of dengue infection.” Surveyors detected dengue patients from among people visiting a local health centre with acute febrile illness and collected blood samples from consenting patients to “examine whether specific characteristics of an urban slum community were associated with the risk of dengue disease. [6]”

Other approaches termed as “community based surveillance”:

- Community members collecting information from the community for research purposes (11%, n=20), e.g. “*community-based surveillance [...] conducted in the 6 randomly selected unions of the intervention arm of the Projahnmo-2 trial*” where community health workers collected information related on neonatal illness through bimonthly home visits to “*assess the population-based incidence and aetiology of community acquired viral pathogens among ill neonate in rural Bangladesh*. [7]”
- Non-community members collecting information from the community for public health surveillance purposes (5%, n=9), e.g. “*community-based surveillance*” system in Vietnam, known as Filabavi demographic surveillance system, surveyors collected data on vital events through home visits for *“monitoring changes in health and function of the health system in a defined community*. [8]”
- Surveyors collecting information from healthcare facility patients for public health surveillance purposes (3%, n=5), e.g. *”community-based influenza surveillance”* local health department collected information from local hospitals on influenza like illness cases for “*early influenza detection.* [9]*”*

References

1. R Core Team. R: A language and environment for statistical computing. Vienna: R Foundation for Statistical Computing, 2017. Available from: https://www.R-project.org/.

2. Vanek MJ, Shoo B, Mtasiwa D, Kiama M, Lindsay SW, Fillinger U, et al. Community-based surveillance of malaria vector larval habitats: a baseline study in urban Dar es Salaam, Tanzania. BMC Public Health. 2006;6: 154.

3. Webb BJ, Hauck FR, Houp E, Portaels F. Buruli ulcer in West Africa: strategies for early detection and treatment in the antibiotic era. East Afr J Public Health. 2009;6: 144–7.

4. Oum S, Chandramohan D, Cairncross S. Community-based surveillance: a pilot study from rural Cambodia. Trop Med Int Health. 2005;10: 689–97.

5. Calva JJ, Sifuentes-Osornio J, Cerón C. Antimicrobial resistance in fecal flora: longitudinal community-based surveillance of children from urban Mexico. Antimicrob Agents Chemother. 1996;40: 1699–702.

6. Kikuti M, Cunha GM, Paploski IAD, Kasper AM, Silva MMO, Tavares AS, et al. Spatial Distribution of Dengue in a Brazilian Urban Slum Setting: Role of Socioeconomic Gradient in Disease Risk. PLoS Negl Trop Dis. 2015;9: e0003937.

7. Farzin A, Saha SK, Baqui AH, Choi Y, Ahmed NU, Simoes EAF, et al. Population-based Incidence and Etiology of Community-acquired Neonatal Viral Infections in Bangladesh: A Community-based and Hospital-based Surveillance Study. Pediatr Infect Dis J. 2015;34: 706–11.

8. Chuc NTK, Diwan VK. FilaBavi, a demographic surveillance site, an epidemiological field laboratory in Vietnam. Scand J Public Health. 2003;31: 3–7.

9. Ghosh TS, Vogt RL. Active influenza surveillance at the local level: A model for local health agencies. Am J Public Health. 2008;98: 213–5.
